# The Differential Role of Central and Bridge Symptoms in Deactivating Psychopathological Networks

**DOI:** 10.3389/fpsyg.2019.02448

**Published:** 2019-11-01

**Authors:** Daniel Castro, Filipa Ferreira, Inês de Castro, Ana Rita Rodrigues, Marta Correia, Josefina Ribeiro, Tiago Bento Ferreira

**Affiliations:** ^1^Department of Social and Behavioural Sciences, University Institute of Maia, Maia, Portugal; ^2^Center for Psychology at University of Porto, Porto, Portugal

**Keywords:** vulnerability, central symptoms, bridge symptoms, psychopathology, network analysis

## Abstract

The network model of psychopathology suggests that central and bridge symptoms represent promising treatment targets because they may accelerate the deactivation of the network of interactions between the symptoms of mental disorders. However, the evidence confirming this hypothesis is scarce. This study re-analyzed a convenience sample of 51 cross-sectional psychopathological networks published in previous studies addressing diverse mental disorders or clinically relevant problems. In order to address the hypothesis that central and bridge symptoms are valuable treatment targets, this study simulated five distinct attack conditions on the psychopathological networks by deactivating symptoms based on two characteristics of central symptoms (degree and strength), two characteristics of bridge symptoms (overlap and bridgeness), and at random. The differential impact of the characteristics of these symptoms was assessed in terms of the magnitude and the extent of the attack required to achieve a maximum impact on the number of components, average path length, and connectivity. Only moderate evidence was obtained to sustain the hypothesis that central and bridge symptoms constitute preferential treatment targets. The results suggest that the degree, strength, and bridgeness attack conditions are more effective than the random attack condition only in increasing the number of components of the psychopathological networks. The degree attack condition seemed to perform better than the strength, bridgeness, and overlap attack conditions. Overlapping symptoms evidenced limited impact on the psychopathological networks. The need to address the basic mechanisms underlying the structure and dynamics of psychopathological networks through the expansion of the current methodological framework and its consolidation in more robust theories is stressed.

## Introduction

**FIGURE 1:**
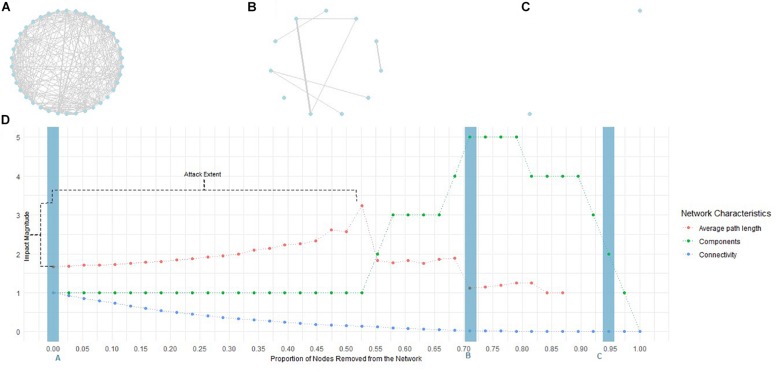
Illustrative example of network attack. Symptom networks are usually represented in a graph comprised of nodes representing symptoms (blue circles in **A–C**) and edges representing the interactions between symptoms (gray lines in **A–C**; line width represents the strength of the interaction). The complete graph represents the causal structure of the interactions between the symptoms. Its exploration provides information regarding the centrality of diverse symptoms (e.g., the number of interactions with other symptoms), as well as the network characteristics (e.g., the number of symptoms, or groups of symptoms, disconnected from the remainder). **(A)** Represents the original psychopathological network. Under attack, the symptoms (nodes) were sequentially removed, one at a time, in the decreasing order of two centrality measures – (1) degree and (2) strength – and two bridging measures – (3) bridgeness and (4) overlap. **(B,C)** Represent the evolution of the initial network as symptoms are removed. At each point, network characteristics were measured (average path length, number of components, connectivity, and diameter). The evolution of these measures throughout the attack is represented in **(D)**. The blue rectangles signal the moments to which the networks represented in **(A–C)** correspond. Finally, the attack extent and the magnitude of its impact were measured considering the number of symptoms that had to be removed to achieve peak average path length, number of components, connectivity, and diameter, as well as the difference between peak and initial values.

Traditional models of psychopathology (e.g., categorical and dimensional) continue to display a vast array of limitations, including a lack of explanation for high rates of comorbidity (Kessler et al., 2005; Goldberg, 2015) and the diversity of clinical presentations (Boschloo et al., 2015; Nuijten et al., 2016). They have also been unable to explain the direct interactions between symptoms (Cramer and Borsboom, 2015) and offer poor support for the identification of etiopathogenic mechanisms and biomarkers of mental disorders (Fried and Nesse, 2015). In recent years, the network theory of mental disorders (Cramer et al., 2010; Borsboom and Cramer, 2013; Borsboom, 2017; Borsboom et al., 2019) has received increased attention as an alternative model that may overcome such persistent limitations. Instead of assuming that symptoms are the effects and measures of a latent dimension (psychological, genetic, neurophysiological, or otherwise) at the origin of mental disorders, this perspective theorizes that mental disorders emerge from a complex network of causal interactions between symptoms (Cramer et al., 2010; Hofmann et al., 2016; Borsboom et al., 2019). Figure 1A presents a typical illustration of these psychopathological networks. In it, the nodes (circles) represent symptoms and the edges (lines) connecting the nodes represent the multiple interactions that constitute the causal structure that is the origin of mental disorders (see Epskamp and Fried, 2018 for a brief discussion on the issue of causality in psychopathological networks). On this basis, it is hypothesized (Borsboom, 2017), and current evidence supports this hypothesis (Cramer et al., 2016), that in an asymptomatic state, this structure remains inactive, but as some symptoms are activated, for example by external events, this generates a cascading effect that spreads through the network, activating the other symptoms to which they are connected. As symptom activation spreads following the paths defined by the structure of interactions between symptoms, the symptoms become increasingly connected until the system transitions into a disease state in which the strongly connected network of symptoms sustains itself, even in the absence of the original activating event (Cramer et al., 2016).

This perspective changes the focus from the syndromatic boundaries of mental disorders or their shared causes to the intricate interplay between symptoms. Inspection of the network of interactions between symptoms has resulted in new understandings of the nosography and comorbidity structures of different mental disorders such as, for example, depression and anxiety (Beard et al., 2016), complex post-traumatic stress disorder (Knefel et al., 2019), or borderline personality disorder and bipolar disorder (Castro et al., 2018). To a significant extent, these advances have been made via the identification of central and bridge symptoms (e.g., Robinaugh et al., 2014; Bekhuis et al., 2016; Knefel et al., 2016; Borsboom, 2017; Levinson et al., 2017; Marcus et al., 2018; Smith et al., 2018).

### Central and Bridge Symptoms

Central symptoms are defined and identified through a set of centrality measures, namely degree, strength, closeness, and betweenness (Opsahl et al., 2010). Degree centrality corresponds to the number of connections a symptom has with the other symptoms in a network and strength to the sum of the absolute weights of these connections (Fried et al., 2017; Epskamp et al., 2018a). Closeness refers to the average proximity of a given symptom to all other symptoms, and betweenness measures the number of times a symptom is on the shortest path between two other symptoms (Epskamp et al., 2018a). However, recent research has concluded that closeness and betweenness may be affected by sampling variability and spurious covariance between symptoms (Hallquist et al., 2019) and are not always adequately stable (Epskamp et al., 2018a). It was also concluded that strength centrality might signal the association of a symptom with a latent variable instead of its role in the network (Hallquist et al., 2019). Moreover, the theoretical foundation for centrality measures in psychopathological networks is still limited (Bringmann et al., 2018). This constrains the meaningful interpretation of high-scoring symptoms in these measures, and the determination of central symptoms remains mostly restricted to those identified via degree or strength centralities.

In turn, bridge symptoms are broadly defined as symptoms that connect different clusters of symptoms corresponding to different mental disorders or subgroups of symptoms within the same mental disorder. In the early stages of the field, Cramer et al. (2010) defined bridge symptoms as those symptoms that overlap perfectly between mental disorders, meaning that bridge symptoms are those that belong to the diagnostic criteria of distinct disorders. For example, fatigue belongs to the diagnostic criteria of major depression disorder and generalized anxiety disorder (American Psychiatric Association [APA], 2013) and should therefore constitute a bridge symptom between these two disorders. Although this initial formulation, as well as subsequent studies that have utilized it Borsboom et al. (2011) and Afzali M. et al. (2017), continue to be generative, other conceptualizations of bridge symptoms have emerged. Some authors have defined bridge symptoms as those that connect different disorders, regardless of any overlap between them (e.g., Robinaugh et al., 2014; Levinson et al., 2017; McNally R. J. et al., 2017; Marcus et al., 2018; Smith et al., 2018). For example, Levinson et al. (2017) suggested that symptoms related to physical sensations might constitute the bridge symptoms between bulimia and anxiety because they are the symptoms of anxiety that are connected to the symptoms of bulimia. These different conceptualizations imply two different kinds of bridge symptoms: (1) those that belong to two or more mental disorders (overlapping symptoms), and (2) those that belong to just one disorder, or alternatively are not specific symptoms of a disorder, but nevertheless still play an important role in connecting different disorders (bridging symptoms). Significantly, the conceptual distinction between overlapping and bridging symptoms has a correspondence in the alternative methodological approaches that have addressed the problem of identifying bridge symptoms. Advances in the identification of bridge symptoms have been achieved by exploring the concept of network communities or modules (e.g., McNally R. J. et al., 2017). Network modules (Fortunato, 2010; Fortunato and Hric, 2016) are sets of symptoms that tend to interact more strongly and therefore exert a greater influence on each other than on the rest of the symptoms in the network. In psychopathological networks, these modules correspond to a mental disorder or to subgroups of symptoms within the same disorder. Jones et al. (2018) proposed a set of measures of the bridge centrality of symptoms that were specifically designed to detect and quantify interacting symptoms between modules. They found that deactivating symptoms based on their bridge strength was more effective for preventing symptom activation from spreading than deactivating symptoms based on their strength or betweenness. This provides evidence in support of the theoretical proposal that bridge symptoms are implicated in the emergence of comorbidity structures between mental disorders (Cramer et al., 2010). By assuming that modules do not overlap, this method implies a concept of bridge symptoms that is closer to that of bridging symptoms. To date, studies characterizing the modular structure of psychopathological networks have mostly followed this perspective, as they typically involve module-detection algorithms (for example, walktrapp Price et al., 2019; spin-glass Birkeland and Heir, 2017) that result in non-overlapping modules. Blanken et al. (2018) have assumed an alternative perspective in exploring the usefulness of the Clique Percolation Method (CPM; Palla et al., 2005; Adamcsek et al., 2006) to detect overlapping modules in psychopathological networks. In the CPM, modules may share overlapping symptoms, which means that one symptom can belong simultaneously to more than one module (Blanken et al., 2018), and therefore it endorses a concept of bridge symptoms that coincides with that of overlapping symptoms. On this basis, Blanken et al. (2018) illustrated how the symptoms that communicate between different modules may explain the diversity of possible pathways resulting in the clinical heterogeneity of mental disorders.

Although differences between these two conceptualizations are not always apparent, preliminary evidence suggests that these types of bridge symptoms may perform different functions on psychopathological networks. In a previous study (Castro et al., 2018) on the modularity of the network of bipolar and borderline personality disorders, an alternative algorithm – ModuLand (Kovács et al., 2010) – was used to explore the comorbidity structure of these mental disorders and differentiate the functions of symptoms in that structure. ModuLand also allows the modules to overlap and computes two different measures – modular overlap and modular bridgeness – each of which enables the identification of different types of bridge symptoms. Akin to the proposal of Jones et al. (2018), modular bridgeness focuses on the inter-modular role of symptoms and provides the effective number of modules to which a symptom is connected (Szalay-Beko et al., 2012). In parallel with the proposal of Blanken et al. (2018), modular overlap focuses on the trans-modular role of symptoms and specifies the overlap of a given symptom between two or more modules relative to all other symptoms (Szalay-Beko et al., 2012). In that study, Castro et al. (2018) found a moderate correlation between the strength centrality and modular bridgeness of symptoms but not between strength centrality and modular overlap. This suggests that the distinction between bridging and overlapping symptoms may increase the conceptual clarity of the construct of bridge symptoms and stimulate further theoretical refinement of the network theory of mental disorders.

Despite these initial observations and the potential pathways that they open up for the further development of the network theory on mental disorders, more studies are needed that compare both types of bridge symptoms. In this regard, an integrated framework that provides a coherent rationale for the identification of both types of bridge symptoms is still required. Without it, the theoretical potential of this construct will remain limited. In order to contribute to consolidating an integrated methodological framework for the measurement of bridge symptoms, this study further explores the ModuLand algorithm (Kovács et al., 2010). Kovács et al. (2010) found Moduland to be robust in characterizing the modular network structure over a number of benchmark networks representing different phenomena (social, biological, semantic) and to be more sensitive and also provide more detailed functional specificity than CPM.

### Central and Bridge Symptoms as Psychotherapeutic Targets

As mentioned, central and bridge symptoms play an important part in the analysis of psychopathological networks and are, on the basis of some of the most promising hypotheses, pertinent to treatments of mental disorders that are currently emerging within the field. From the network theory perspective, treatments of mental disorders exert some influence on the network of interactions between symptoms by deactivating symptoms, inhibiting their interactions, or removing external events that trigger activation cascades (see Borsboom, 2017; Isvoranu et al., 2018). Irrespective of the pathway through which treatments exert their influence, psychopathological networks, as they unfold, are expected to follow a trajectory similar to the one depicted in Figures 1A–C. As represented in those figures, inhibition of the interactions between symptoms should be associated with a general decrease in the connectivity of the network (number of interactions present in the network) due to the decrease in the number of active interactions. Similarly, symptom deactivation should also contribute to this decrease, since it also deactivates the interactions in which symptoms are involved. As network connections become sparser, the number of interactions needed to connect the symptoms that remain active is expected to increase (i.e., the pathways between them become longer). Finally, the decrease in network connectivity and the increase in the length of the pathways between symptoms should cause some symptoms to become isolated, and it should therefore be accompanied by an increase in the number of disconnected components. In addition to being theoretically coherent, this proposal is also consistent with routine clinical observations since recovered patients, by definition, report a minimal number of mild residual symptoms to no symptoms at all. It is therefore surprising that studies comparing the connectivity of psychopathological networks at treatment admission and discharge or follow-up have provided mixed evidence for a decrease in the connectivity of the network. Some studies revealed a pattern of network transformation consistent with the one described above, with decreased connectivity at discharge (and, in some samples, isolated symptoms; Snippe et al., 2017), but no statistically significant differences compared to admission (Snippe et al., 2017) and placebo or wait-list control groups (Snippe et al., 2017) were found. A previous study also found higher connectivity in a group of poor-responders to treatment but no statistically significant difference compared to a group of good-responders (Schweren et al., 2018). In addition, other studies observed an increase in connectivity from admission to discharge (Beard et al., 2016; Bos et al., 2018). These studies have proposed a number of possible explanations (see also Fried E. I. et al., 2016) related, for example, to the persistence of an underlying vulnerability to the development of mental disorders associated with network connectivity even after symptoms decrease (Snippe et al., 2017), naturalistic versus controlled (Schweren et al., 2018) or within- versus between-subject (Bos et al., 2018) designs, and response bias arising from the repeated administration of symptom measures and changes in the interpretation of the items in the measure as treatment unfolds (Fried E. I. et al., 2016; Bos et al., 2018). Additionally, some of the previous studies (Beard et al., 2016; Snippe et al., 2017; Bos et al., 2018) are based on complete samples at discharge. These samples are likely to include participants who did not respond to treatment, other participants who responded to treatment but did not recover, participants who deteriorated, and also participants who recovered across treatment. Other studies (Schweren et al., 2018) are based on participants who responded to treatment but for whom it is unclear that they have recovered. Consequently, in some of these studies, post-treatment samples displayed average symptom severity within the clinically relevant realm (Beard et al., 2016; Snippe et al., 2017; Bos et al., 2018; Kraft et al., 2019) or minimal change in relation to admission (Kraft et al., 2019), and studies analyzing clearly recovered participants are still lacking. The lack of a distinction between different therapeutic trajectories may contribute to explain the observation that despite no statistically significant differences being found in network connectivity from pre- to post-treatment, participants whose networks display higher connectivity at admission also display a poorer response to treatment (Smith et al., 2019). It is also increasingly recognized that these studies pose significant challenges (see Terluin et al., 2016). Reports from recovered participants will necessarily be highly skewed and display a restriction of range that affects both network estimation due to deviations from normality (Epskamp et al., 2018a) and comparison due to the impact of differential variance on the network connectivity (Terluin et al., 2016). Although the precise impacts of these (and other) possible explanations remain to be further clarified, it has been recognized that exploration of the assumption that higher connectivity characterizes psychopathological states is warranted (Schweren et al., 2018) and consistent with existing evidence (Snippe et al., 2017), suggesting that it is in fact associated with a transition to a psychopathological state (Cramer et al., 2016). Also, in natural settings, it has been observed that higher connectivity of cross-sectional psychopathological networks is associated with symptom persistence (compared to remittance; van Borkulo et al., 2015; van Rooijen et al., 2018) and that this connectivity is higher in clinical populations than in healthy populations (Santos et al., 2017; Stone et al., 2017), as well as differing between individuals diagnosed with a mental disorder but displaying distinct clinical presentations (DuBois et al., 2017) and course types (Koenders et al., 2015). The analysis of the global connectivity of longitudinal dynamic networks has produced mixed results. Two studies concluded that the networks of individuals diagnosed with mental disorders displayed higher connectivity (Pe et al., 2015; Wichers et al., 2016), but these results may be dependent on the methodological options during data pre-processing and network estimation (de Vos et al., 2017). Another study (Groen et al., 2019) did not observe differences in the connectivity of the dynamic networks of individuals with persisting symptoms compared to individuals displaying symptom remission. However, in this study (Groen et al., 2019), differences between those groups were observed in the structure of interactions between symptoms (network topological structure), suggesting that the role of specific symptoms may be relevant beyond the global connectivity of the network. This is consistent with the results of other studies that observed changes in the network structure but not in its global connectivity after a brief intervention in remitted symptoms (Kraft et al., 2019) and underlined that treatments impact some specific symptoms (Blanken et al., 2019; Mullarkey et al., 2019). Similar results were obtained when comparing healthy individuals or community-based samples with individuals diagnosed with mental disorders or other medical conditions, both on dynamic (Curtiss et al., 2019) and cross-sectional (Hartung et al., 2019; Montazeri et al., 2019; Silk et al., 2019) networks; and also the cross-sectional networks of individuals displaying low versus high behavioral risk for medical conditions (Choi et al., 2017). Together, these results suggest that the specific role each symptom plays within the network (as defined by the centrality and bridgeness of the symptom) may carry particular significance independently of the global connectivity of the network. This is consistent with an early hypothesis within this research field that central and bridge symptoms constitute priority therapeutic targets (Cramer et al., 2010). Central symptoms were hypothesized to be responsible for maintaining mental disorders, as they are involved in stronger interactions or in the majority of interactions that constitute psychopathological networks (Borsboom and Cramer, 2013). For this reason, it has often been proposed that these symptoms can provide valuable psychotherapeutic targets because they may accelerate the deactivation of the network and consequently catalyze treatments (e.g., Borsboom and Cramer, 2013; McNally et al., 2015; Bekhuis et al., 2016; Knefel et al., 2016; Robinaugh et al., 2016; Bryant et al., 2017; Martel et al., 2017; Richetin et al., 2017; Goldschmidt et al., 2018; Olatunji et al., 2018; Montazeri et al., 2019; see also Fried et al., 2017 for a general overview). Simultaneously, bridge symptoms were hypothesized to be associated with the emergence of comorbidity structures (Cramer et al., 2010; Borsboom, 2017) that are known to hamper the progress of a treatment (Kessler et al., 2005). Accordingly, as with central symptoms, bridge symptoms are considered important treatment targets because the deactivation of these symptoms might prevent the development of comorbidity between mental disorders (e.g., Cramer et al., 2010; Afzali M. et al., 2017; Borsboom, 2017; Choi et al., 2017; DuBois et al., 2017; Levinson et al., 2017; Jones et al., 2018; Rouquette et al., 2018; Garabiles et al., 2019; Solmi et al., 2019). Studies that specifically addressed symptom centrality have noted that it predicts changes in the remaining symptoms (Robinaugh et al., 2016), and facilitates evolution to a psychopathological condition (Boschloo et al., 2016b), whereas others did not find evidence to support the hypothesis that symptom centrality, estimated from cross-sectional networks, is associated with changes in symptoms over time (Bos et al., 2017). To date, the study to most closely test this hypothesis was conducted by Rodebaugh et al. (2018), who explored whether central symptoms in a cross-sectional network of social anxiety disorder predicted changes in symptoms across treatment in another sample of individuals who undertook treatment for the same disorder. They found only moderate support for this hypothesis, as the symptom centrality was not generalized across measures and the frequency of symptom endorsement also predicted change and was generalized across measures of social anxiety disorder. Therefore, the initial hypothesis remains open, and its evaluation will contribute to an increase in the utility and validity of psychopathological networks for routine practice (see Contreras et al., 2019). In this paper, we offer an alternative perspective by focusing on the actual impact of symptom deactivation. In order to deepen our comprehension of the mechanisms that promote the deactivation of psychopathological networks, this study simulates symptom deactivation by removing symptoms from the network according to different regimes dependent on various centrality measures and their roles as bridge symptoms (for studies following similar modeling methods, see Robinaugh et al., 2016; Afzali M. H. et al., 2017; Jones et al., 2018). As is typical of network science, we evaluated the impact of the removal of these symptoms on the global properties of the networks compared with the removal of random symptoms (e.g., Albert et al., 2000; Latora and Marchiori, 2001; Costa et al., 2007) in order to explore the differential efficacy of central and bridge symptoms in deactivating psychopathological networks.

## Materials and Methods

### Networks

In order to collect a sample of networks from the published literature, the databases PsychInfo, Web of Knowledge, Academic Search Complete, and Google Scholar were searched for studies addressing cross-sectional networks of symptoms of mental disorders or other clinically relevant problems (e.g., hopelessness, alexithymia) using a combination of keywords including the names of mental disorders and “psychopathological networks” or “network analysis.” Studies addressing the personality structure or other psychological phenomena that are not directly related to mental disorders or clinically relevant problems were not included. Studies meeting the above criteria were checked for supporting data availability. An initial sample of 34 networks was collected. Seventeen additional networks were included, having been made available in a previous review by Haslbeck and Fried (2017). A convenience sample of 51 cross-sectional networks from 36 previous studies addressing mental disorders or clinically relevant problems was reanalyzed. These networks and the original studies are presented in Supplementary Materials. They are also identified with an asterisk in the reference list.

From the 51 networks, 19 (37.3%) pertained to clinical samples and the remainder to community-based samples; 18 (35.3%) included symptoms from different mental disorders or psychological problems. The network descriptives are presented in Table 1, and the distributions of the network characteristics are summarized in Figure 2.1.

**TABLE 1:** Network descriptives.

**Network characteristics**	**Networks (*N* = 51)**		
	
	***M (SD)***	**M_trimmed_ (SE)**	**Minimum–maximum**
Nodes	22.725 (20.576)	17.419 (1.335)	5.000–120.000
Edges	99.392 (129.186)	65.645 (8.492)	8.000–756.000
Density	0.475 (0.221)	0.485 (0.035)	0.067–0.861
Components	1.333 (0.973)	1.000 (0.000)	1.000–5.000
Average path length	1.658 (0.454)	1.544 (0.053)	1.139–3.283

**FIGURE 2:**
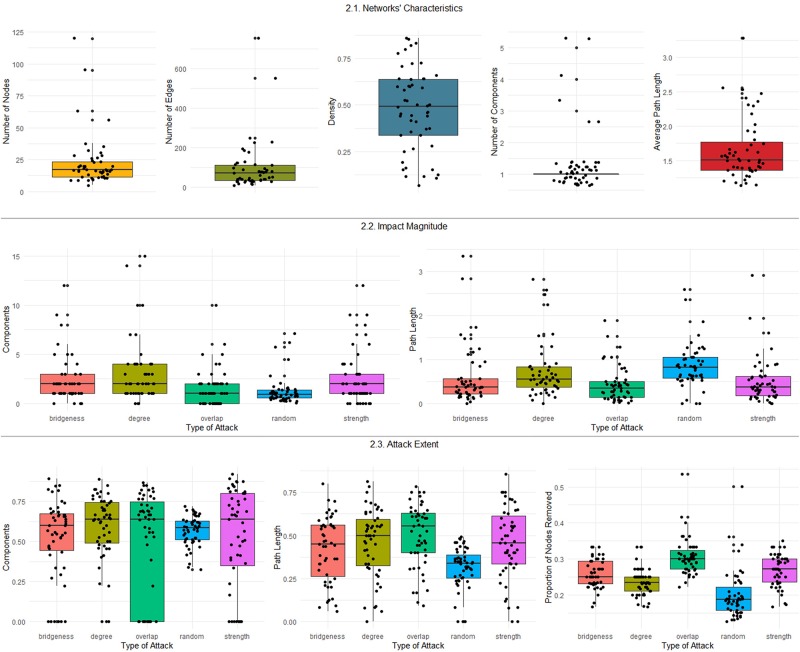
Boxplots summarizing the distributions of the network characteristics, impact magnitude, and attack extent.

### Data Analyses

Originally, networks were estimated using correlation matrices (e.g., Goekoop and Goekoop, 2014; Robinaugh et al., 2014; Koenders et al., 2015), partial correlation matrices (e.g., Anderson et al., 2015), the PC algorithm (Ruzzano et al., 2015), Gaussian Graphical Models (GGM; e.g., Fried E. et al., 2016; Fried et al., 2018), or Ising models (e.g., Boschloo et al., 2015; Kendler et al., 2017). Following current standards in psychopathological network estimation (Epskamp et al., 2018a), where raw data or correlation matrixes were available, networks were re-estimated using GGM (Epskamp and Fried, 2018) or Ising models (van Borkulo et al., 2014) in the case of continuous and binary data, respectively. GGM implemented in the qgraph package (version 1.5; Epskamp et al., 2012) for R (version 3.5.1; R Core Team, 2019) represents the most widely used method for estimating psychopathological networks from continuous data. This model estimates a network of partial correlation coefficient (Epskamp et al., 2018b). The Ising model is based on logistic regressions and is a commonly used procedure for estimating networks from binary data (van Borkulo et al., 2014). This model is implemented through the IsingFit package (version 0.3.1; van Borkulo et al., 2014) for R (version 3.5.1; R Core Team, 2019). Both methods use the Least Absolute Shrinkage and Selection Operator (LASSO) regularization technique (Tibshirani, 1996) in order to control for spurious interactions and the Extended Bayesian Information Criterion (EBIC; Chen and Chen, 2008) for model selection (see Epskamp et al., 2018a for a detailed discussion of these methods for psychopathological network estimation).

Where previous studies have analyzed different networks estimated from the same sample (e.g., DuBois et al., 2017; Santos et al., 2017; Smith et al., 2018), only one was included in the current study. Analyses were based on the adjacency matrixes provided by the original authors or by Haslbeck and Fried (2017) when raw data or correlation matrixes were unavailable.

The graphical representation of the networks was performed using the qgraph package (version 1.5; Epskamp et al., 2012) for R (version 3.5.1; R Core Team, 2019). The measures of symptom centrality, namely degree and strength, were also computed using the qgraph package. Degree corresponds to the number of connections a symptom has with the other symptoms. Strength corresponds to the sum of the weight of all connections from one symptom to the remainder. These measures were selected because they are consistently stable across studies, unlike other measures like betweenness and closeness, which are frequently unstable (Epskamp et al., 2018a).

The ModuLand algorithm (Szalay-Beko et al., 2012), implemented in Cytoscape 3.5.1 (Shannon et al., 2003), was used to measure the two types of bridge symptoms discussed above. ModuLand allocates each symptom to a module based on assignment values that represent how much a symptom belongs to each module. Thus, this framework permits the identification of modular overlap, which is a trans-modularity measure of the number of modules to which a symptom is assigned, and modular bridgeness, an inter-modularity measure of the effective number of modules to which a symptom is connected (Szalay-Beko et al., 2012).

In order to explore the characteristics of the deactivation of psychopathological networks, symptom deactivation was simulated by removing symptoms successively, one at a time, and the network characteristics were measured after the removal of each symptom (see Barabási, 2016; also see Albert et al., 2000; Latora and Marchiori, 2001; Mishkovski et al., 2011 for examples of the same procedure in different domains of network science). The differential impact of the central and bridge symptoms was examined by simulating five attack conditions. Symptoms were removed in the decreasing order of (1) degree, (2) strength, (3) bridgeness, and (4) overlap. These were also compared to a fifth condition, in which symptoms were randomly removed from the network. This was performed using the ProNet package (version 1.0.0; Wu and Xia, 2015) for R (version 3.5.1; R Core Team, 2019). The number of simulations for the random attack was set at 2,000. At each time, three characteristics of the networks were measured: connectivity, components, and average path length. The igraph package (version 1.2.2; Csárdi and Nepusz, 2006) for R (version 3.5.1; R Core Team, 2019) was used to compute the characteristics of the network. Connectivity measures the sum of the number of interactions between all symptoms in the network. Components refer to portions (symptoms or groups of symptoms) of the network that are disconnected from the rest of the network. Finally, the average path length is the mean of the shortest paths in the network (Costa et al., 2007).

The impact generated by each attack condition was measured by computing the magnitude of the impact and the extent of the attack required to achieve maximum impact. First, we computed the difference between the average path length and the maximum value of the number of components and the initial values displayed by the network. Second, the proportion of symptoms that had to be removed to achieve the maximum average path length and number of components was computed. Finally, the impact on network connectivity was measured by computing the proportion of symptoms that needed to be removed to achieve a 50% drop in network connectivity. This procedure is illustrated in Figure 1.

The analysis of the distributions of the characteristics of the networks, impact magnitude, and attack extent (summarized in Figures 2.2, 2.3) revealed high dispersion, extreme values, and skewed distributions. For this reason, the robust bootstrap-t method proposed by Wilcox (2017; see also Field and Wilcox, 2017 for an introduction to this method) for comparing multiple dependent trimmed means was used to compare the effect of the attack conditions on the magnitude of the impact as well as the extent of the attack on the number of components, average path length, and connectivity. The robust test includes the test statistic and the critical value for the test statistic (at α = 0.05). Robust *post hoc* tests display the difference between trimmed means (psihat), its bootstrap 95% confidence interval, the test statistics for this difference, and the critical value for the test. Psihat is negative if the trimmed mean for the first element in the comparison (for example, the trimmed mean number of components in the random versus degree attack conditions) is lower than the trimmed mean for the second element and is positive if it is higher. The test is considered statistically significant (*p* < 0.05) if the test statistic exceeds the critical value. The amount of trim in computing trimmed means was set at 0.2, and 2,000 bootstrap samples were considered. The R package WRS2 (version 0.10-0; Mair and Wilcox, 2018) was used to compute robust statistics. All data analysis on R was performed in RStudio 1.1.379 (RStudio Team, 2017). R-code is available from the corresponding author upon request.

## Results

The extent of the attack and the magnitude of its impact under the different attack conditions are presented in Table 2. (The evolution of the characteristics of each network under the different attack conditions is included in the Supplementary Materials). The distributions of the attack extent and impact magnitude for the different attack conditions are summarized in Figures 2.2, 2.3.

**TABLE 2:** Descriptives of impact magnitude and attack extent.

**Network characteristics/attack condition**	**Impact magnitude**			**Attack extent**		
		
	***M (SD)***	**M_trimmed_ (SE)**	**Minimum–maximum**	**M (SD)**	**M_trimmed_ (SE)**	**Minimum–maximum**
**Components**						
Random	1.300 (1.469)	0.926 (0.089)	0.060–7.096	0.568 (0.093)	0.577 (0.013)	0.323–0.718
Degree	3.098 (3.183)	2.290 (0.295)	0.000–15.000	0.587 (0.213)	0.623 (0.027)	0.000–0.889
Strength	2.471 (2.701)	1.806 (0.291)	0.000–12.000	0.523 (0.316)	0.582 (0.058)	0.000–0.921
Bridgeness	2.412 (2.401)	1.774 (0.198)	0.000–12.000	0.527 (0.245)	0.574 (0.032)	0.000–0.893
Overlap	1.608 (1.866)	1.194 (0.190)	0.000–10.000	0.493 (0.327)	0.545 (0.074)	0.000–0.868
**Path length**						
Random	0.855 (0.502)	0.801 (0.054)	0.000–2.588	0.315 (0.119)	0.326 (0.016)	0.000–0.493
Degree	0.735 (0.629)	0.576 (0.052)	0.000–2.820	0.459 (0.200)	0.482 (0.031)	0.000–0.812
Strength	0.511 (0.521)	0.391 (0.052)	0.000–2.904	0.454 (0.208)	0.467 (0.031)	0.000–0.857
Bridgeness	0.575 (0.650)	0.382 (0.057)	0.001–3.345	0.420 (0.191)	0.436 (0.034)	0.059–0.800
Overlap	0.431 (0.398)	0.333 (0.041)	0.010–1.879	0.503 (0.182)	0.526 (0.028)	0.091–0.786
Connectivity						
Random	–	–	–	0.201 (0.067)	0.187 (0.007)	0.127–0.501
Degree	–	–	–	0.236 (0.036)	0.235 (0.005)	0.167–0.333
Strength	–	–	–	0.270–0.044	0.275 (0.007)	0.167–0.350
Bridgeness	–	–	–	0.260 (0.039)	0.259 (0.007)	0.167–0.333
Overlap	–	–	–	0.306 (0.051)	0.300 (0.006)	0.222–0.536

The robust bootstrap-t method for comparing multiple dependent means was conducted to compare the effect of the attack conditions on the magnitude of the impact on the number of components and average path length in the network. A statistically significant difference was observed between the attack conditions on the trimmed mean impact on the number of components, *F*_*t*_ = 14.514, *F*_*crit*_ = 2.499, *p* < 0.05. The *post hoc* comparisons between attack conditions reported in Table 3 suggest that the degree, strength, and bridgeness attack conditions (but not overlap attack) yielded a significantly higher impact than the random attack on the number of components. No significant differences were observed between these three conditions as to the magnitude of the impact on the number of components. Degree and bridgeness attack conditions also yielded a significantly higher impact than overlap attack on the number of components. A statistically significant difference between attack conditions on the trimmed mean impact on the average path length was also observed, *F*_*t*_ = 28.790, *F*_*crit*_ = 2.387, *p* < 0.05. The *post hoc* comparisons between attack conditions, reported in Table 4, suggest that the random attack condition had a higher impact on the average path length than the remaining attack conditions; moreover, degree attack had a stronger impact on the average path length than the strength, bridgeness, and overlap conditions.

**TABLE 3:** *Post hoc* comparisons between attack conditions of the magnitude of the impact on the number of components.

**Pairwise comparisons**	**psihat**	**95% CI**	**Test statistics**	**Critical value of the test**
Random vs. Degree	–1.364	[−2.124, −0.604]	−5.624^∗^	3.132
Random vs. Strength	–0.880	[−1.627, −0.134]	−3.694^∗^	3.132
Random vs. Bridgeness	–0.848	[−1.330, −0.366]	−5.513^∗^	3.132
Random vs. Overlap	–0.2671	[−0.787, 0.253]	–1.610	3.132
Degree vs. Strength	0.484	[−0.084, 1.052]	2.668	3.132
Degree vs. Bridgeness	0.516	[−0.015, 1.047]	3.044	3.132
Degree vs. Overlap	1.097	[0.373, 1.821]	4.745^∗^	3.132
Strength vs. Bridgeness	0.032	[−0.572, 0.637]	0.167	3.132
Strength vs. Overlap	0.613	[−0.098, 1.324]	2.700	3.132
Bridgeness vs. Overlap	0.581	[0.022, 1.140]	3.250^∗^	3.132

*^∗^Statistically significant, *p* < 0.05.*

**TABLE 4:** *Post hoc* comparisons between attack conditions of the magnitude of the impact on the average path length.

**Pairwise comparisons**	**psihat**	**95% CI**	**Test statistics**	**Critical value of the test**
Random vs. Degree	0.225	[0.079, 0.372]	4.346^∗^	2.826
Random vs. Strength	0.410	[0.250, 0.570]	7.242^∗^	2.826
Random vs. Bridgeness	0.419	[0.260, 0.579]	7.437^∗^	2.826
Random vs. Overlap	0.468	[0.312, 0.625]	8.447^∗^	2.826
Degree vs. Strength	0.185	[0.038, 0.332]	3.554^∗^	2.826
Degree vs. Bridgeness	0.194	[0.065, 0.323]	4.237^∗^	2.826
Degree vs. Overlap	0.243	[0.090, 0.396]	4.479^∗^	2.826
Strength vs. Bridgeness	0.009	[−0.122, 0.140]	0.195	2.826
Strength vs. Overlap	0.058	[−0.078, 0.194]	1.202	2.826
Bridgeness vs. Overlap	0.049	[−0.068, 0.166]	1.186	2.826

*^∗^Statistically significant, *p* < 0.05.*

The robust bootstrap-t method for comparing multiple dependent means was also utilized to compare the effect of the attack conditions on the extent of the attack required to achieve maximum impact on the number of components, average path length, and connectivity of the network. A non-statistically significant difference was observed between the trimmed mean extent of the attack on the number of components between attack conditions, *F*_*t*_ = 0.446, *F*_*crit*_ = 3.912, *p* > 0.05. A statistically significant difference was observed between the trimmed mean extent of the attack on the average path length between attack conditions, *F*_*t*_ = 17.752, *F*_*crit*_ = 2.511, *p* < 0.05. The *post hoc* comparisons between attack conditions, reported in Table 5, suggest that the random attack performs better than all of the remaining attack conditions in extending the average path length. A statistically significant difference between attack conditions on the trimmed mean extent of the attack on the network connectivity was observed, *F*_*t*_ = 57.293, *F*_*crit*_ = 2.808, *p* < 0.05. The *post hoc* comparisons between attacks, reported in Table 6, suggest that the random attack condition performed better than any of the remaining attack conditions in reducing the network connectivity. They also suggest that degree attack performed better than strength, bridgeness, and overlap conditions and that bridgeness attack performed better than overlap attack.

**TABLE 5:** *Post hoc* comparisons between attack conditions of the extent of the attack on the average path length in the network.

**Pairwise comparisons**	**psihat**	**95% CI**	**Test statistics**	**Critical value of the test**
Random vs. Degree	–0.155	[−0.215, −0.096]	−7.702^∗^	2.970
Random vs. Strength	–0.141	[−0.204, −0.078]	−6.618^∗^	2.970
Random vs. Bridgeness	–0.110	[−0.189, −0.030]	−4.111^∗^	2.970
Random vs. Overlap	–0.200	[−0.270, −0.130]	−8.498^∗^	2.970
Degree vs. Strength	0.015	[−0.052, 0.081]	0.655	2.970
Degree vs. Bridgeness	0.046	[−0.024, 0.115]	1.953	2.970
Degree vs. Overlap	–0.045	[−0.123, 0.034]	–1.686	2.970
Strength vs. Bridgeness	0.031	[−0.044, 0.106]	1.220	2.970
Strength vs. Overlap	–0.059	[−0.148, 0.030]	–1.972	2.970
Bridgeness vs. Overlap	–0.090	[−0.181, 0.001]	–2.943	2.970

*^∗^Statistically significant, *p* < 0.05.*

**TABLE 6:** *Post hoc* comparisons between attack conditions of the impact of the extent of the attack on network connectivity.

**Pairwise comparisons**	**psihat**	**95% CI**	**Test statistics**	**Critical value of the test**
Random vs. Degree	–0.049	[−0.075, −0.022]	−5.506^∗^	3.017
Random vs. Strength	–0.088	[−0.122, −0.054]	−7.894^∗^	3.017
Random vs. Bridgeness	–0.073	[−0.101, −0.044]	−7.711^∗^	3.017
Random vs. Overlap	–0.114	[−0.135, −0.092]	−16.200^∗^	3.017
Degree vs. Strength	–0.039	[−0.059, −0.020]	−6.015^∗^	3.017
Degree vs. Bridgeness	–0.024	[−0.041, −0.008]	−4.422^∗^	3.017
Degree vs. Overlap	–0.065	[−0.084, −0.046]	−10.289^∗^	3.017
Strength vs. Bridgeness	0.015	[−0.005, 0.035]	2.298	3.017
Strength vs. Overlap	–0.026	[−0.054, 0.003]	–2.740	3.017
Bridgeness vs. Overlap	–0.041	[−0.065, −0.016]	−5.026^∗^	3.017

*^∗^Statistically significant, *p* < 0.05.*

## Discussion

The network theory of psychopathology has suggested that central and bridge symptoms might constitute priority therapeutic targets due to their ability to accelerate the deactivation of the network of interactions between symptoms (Cramer et al., 2010; Borsboom and Cramer, 2013; Borsboom, 2017). This hypothesis had a strong impact on the field, and it is common for studies using cross-sectional networks to detail the nosographic structure of mental disorders to conclude that the most central or bridge symptoms in those networks could constitute important therapeutic targets. However, few studies have directly tested this hypothesis, and the actual impact of manipulating central and bridge symptoms on the psychopathological networks remains unclear. Mapping this impact will have important consequences for the field, as it may support current innovations in diverse domains, such as treatment personalization (see, e.g., Fisher et al., 2019), and therefore contribute to increasing the relevance of psychopathological networks to routine practice, which has been recognized to remain limited (Contreras et al., 2019; see also Bringmann and Eronen, 2018). In order to contribute to this effort, this study compared the impact of deactivating symptoms (as is assumed to occur during treatment; Borsboom, 2017) according to different conditions corresponding to the different definitions of bridge symptoms and the most common measures of symptom centrality. Given that, in the case of bridge symptoms, different definitions coexist in the literature, we explored an alternative method for their identification based on the modular properties of psychopathological networks. Through ModuLand (Kovács et al., 2010), it was possible to specify both the inter-modular and trans-modular roles that bridge symptoms have been proposed to play in psychopathological networks, which may offer a framework for future studies that directly compare these roles of bridge symptoms.

Globally, the results suggest that attacks based on bridge and central symptoms are less proficient than random attacks in transforming the path length and connectivity of the network but tend (with the exception of overlapping symptoms) to generate a higher number of isolated symptoms. They also suggest that symptoms with the highest degree are more efficient (than symptoms with the highest strength, bridgeness, or overlap) in increasing the path lengths and in decreasing the connectivity of the network. In contrast, overlapping symptoms had the smallest impact on the network. As in previous studies (Rodebaugh et al., 2018), these results provide only moderate evidence in support of the hypothesis that central and bridge symptoms constitute priority treatment targets and are consistent with the observation that common centrality measures (degree, strength, closeness, and betweenness) are only weakly correlated with the causal influence of symptoms on the network (Dablander and Hinne, 2018).

However, they also add to a small number of studies (Robinaugh et al., 2016; Dablander and Hinne, 2018) that converge in highlighting that distinct centrality measures perform differently and that there are measures of network operation and symptom centrality other than those most commonly used in the field that constitute relevant alternatives. For example, psychopathological networks have been explored with emphasis placed on the global connectivity of the network, such as through comparisons between pre-treatment and post-treatment connectivity (Beard et al., 2016; Snippe et al., 2017; Bos et al., 2018). Although results from these studies are not always consistent (e.g., van Borkulo et al., 2015; Bos et al., 2018), the suggestion remains that network connectivity is the prime property for detecting changes in networks. This is grounded in strong theoretical assumptions (Borsboom, 2017) and empirical evidence (Cramer et al., 2016), but the results from this study suggest that other characteristics of psychopathological networks should be further explored, since changes in the number of components appear to be easier to accomplish with targeted attacks (guided by symptom degree, strength, and bridgeness), and therefore this might represent a better indicator for detecting changes in psychopathological networks. Nonetheless, the number of components is an unexplored measure of psychopathological networks, and the meaning of these changes does not currently have a theoretical foundation to enable us to derive a decisive conclusion regarding its importance for psychopathological networks. In fact, in most cases, after a successful therapeutic process, the remission of symptoms is not total (see Rottenberg et al., 2018 for an example in depression), so it is fair to assume that these symptoms might still be forming separate components. Connectivity within these components might still be high, so the change in connectivity is not particularly noticeable, and an increase in the number of components might be a better measure for therapeutic success.

Another example stems from the finding that attacks based on symptoms with higher overlap do not produce significant changes in the characteristics of the network, in contrast to the other attack conditions. This is consistent with another study (Castro et al., 2018) that reported a moderate correlation between symptom strength and bridgeness but not between strength and overlap. Although this suggests that overlapping symptoms may not be of special relevance to inform developments in psychotherapeutic treatments based on psychopathological networks, since they do not seem to pinpoint a vulnerability of these networks, it does not exclude the hypothesis that they may play an important role in their constitution. In fact, recent studies have proposed methods for the identification of these symptoms on the basis that they might be more akin to the nature of psychopathological processes (Blanken et al., 2018). The two types of bridge symptoms may therefore play distinctive roles on the psychopathological networks, and future research should aim to provide further knowledge on the roles of bridging and overlapping symptoms. It is reasonable to hypothesize, since symptoms displaying higher modular bridgeness also seem to display stronger connections to other symptoms in the network, that bridging symptoms may be more involved in aggregating symptoms and maintaining the integrity of the network of interactions between them than the overlapping symptoms. If this is the case, then bridging symptoms may be implicated in the vulnerability of a network to transition to a mental disorder state since vulnerability is associated with stronger connectivity (Cramer et al., 2016) and bridge symptoms may regulate the flux of information in psychopathological networks (Jones et al., 2018). On the other hand, it has been suggested that overlapping elements in complex networks that have few and weak links to other elements but connect nodes in distinct regions of the network contribute to the flexibility and adaptability of the network (Csermely, 2008); this may help to explain the variability of the clinical presentations and subtypes of mental disorders (e.g., Galatzer-Levy and Bryant, 2013; Fried and Nesse, 2015). If the different functions of bridging and overlapping symptoms are confirmed in future studies, it would add to that general hypothesis the possibility that they contribute to the constitution of comorbidity structures through distinct mechanisms: bridging symptoms by connecting otherwise distinct modules from distant regions of the network and overlapping symptoms by creating a shared vulnerability for the synchronized activation of several modules.

The type of attack is another important aspect of this study. We have only computed the symptom centrality and bridge measures for the initial network, and the order for the symptom removal was established based on this first estimation. This approach was used because most research identifies the central and bridge symptoms and proposes them as therapeutic targets based only on a single time-point estimation of the network. However, considering the particularities of psychopathological networks, such as the high density and small number of symptoms, this might not be the most appropriate approach to modeling symptom deactivation. In some cases, these characteristics of psychopathological networks result in the removal of a symptom without the ability to meaningfully affect the characteristics of the network. If all of the highest-scoring symptoms have high degree values mostly due to the connections between them, the removal of these symptoms based on the first estimation will arrive at a point where the symptom being removed is no longer relevant for the network. Given that removing one symptom also removes the existing connections, with the symptoms being largely interconnected, their removal after the initial attack leaves the remaining network unaffected. In these cases, the random attack has a higher probability of removing a more connected symptom after the initial attack. In order to overcome this limitation, future research should test other types of targeted attacks, such as cascading attacks (Motter and Lai, 2002; Wang and Rong, 2009). In a cascading attack, symptom centrality is re-estimated after the deactivation of each symptom. Consequently, at each step, the symptom with the highest centrality reflects the network state at that point and not the initial state of the network. These attacks are more harmful to certain networks (Holme et al., 2002), and, in light of the results of this study, this may be a more appropriate strategy for exploring the vulnerability of the network.

Another limitation of this study is that it tested a restricted range of centrality measures. We have excluded two commonly used measures in psychopathological networks (betweenness and closeness) based on the lack of stability they have exhibited in psychopathological networks (Epskamp et al., 2018a) and opted for the inclusion of only the most frequently used centrality measures in the field. This was also done in favor of better consistency and interpretability of the results. However, other measures of symptom impact have emerged within the field (Robinaugh et al., 2016; Jones, 2018), and network science offers a vast array of centrality measures (e.g., Wang et al., 2014) that might be generative in the study of the vulnerability of psychopathological networks but remain unexplored. This suggestion to use alternative centrality measures has recently been proposed by Bringmann et al. (2018). It is consistent with the findings that alternative centrality measures (e.g., eigenvector centrality) reveal higher correlations with the causal role of symptoms than the most common measures (Dablander and Hinne, 2018) and that these common measures may not adequately account for some of the characteristics of psychopathological networks (e.g., the negative weights of some of the connections between symptoms; Robinaugh et al., 2016). The examination of alternative measures is necessary for the continued development of the network theory of psychopathology, but it should not be done in a random way. Evidence from other fields suggests that the nature of the network dictates its specific vulnerability to different types of attacks (Holme et al., 2002) and the most relevant centrality measures (Borgatti, 2005). An initial study (Borsboom et al., 2011) explored the topological structure of the network of DSM-IV (American Psychiatric Association [APA], 1994) symptoms, concluding that it is consistent with the characteristics of a general type of complex network termed a small-world network (Watts and Strogatz, 1998), meaning that most symptoms, even those from mental disorders traditionally considered distant, are connected by short paths. This was offered as an explanation to the pervasiveness of comorbidity between mental disorders and the unsuccessful search for causal mechanisms and biomarkers. No further attempts were made to replicate this study, and most of the recent networks in the field refer to smaller sets of symptoms pertaining to specific mental disorders. Therefore, the characteristics of the topological structure of psychopathological networks remain largely unknown. As these characteristics, as well as the specific processes unfolding within the network, determine the most relevant measures of its operation (Borgatti, 2005), increased efforts to clarify the characteristics of the topological structure of psychopathological networks could guide the principled identification of the most relevant centrality measures for psychopathological networks. It could also contribute to the study of the vulnerability of psychopathological networks and advance the identification of the characteristics that may more productively be explored by psychotherapeutic treatments.

It should also be noted that all of the networks used in this study were based on cross-sectional data, which has been pointed out as a limitation in the identification of central symptoms (Robinaugh et al., 2014; Fried et al., 2015; van Borkulo et al., 2015). In fact, previous studies found differences in the central symptoms of cross-sectional and of longitudinal networks (e.g., Bos et al., 2017; see also Contreras et al., 2019 for a review). In this study, cross-sectional networks were used because, to date, research suggesting central and bridge symptoms are important therapeutic targets is mostly based on cross-sectional networks. However, replication studies based on longitudinal networks are needed.

Finally, this study examined a convenience sample of previously published data sets that encompass different mental disorders. Its findings aggregate all of the networks, potentially resulting in the omission of specific mechanisms associated with the differential vulnerabilities of distinct mental disorders. Whether the psychopathological networks characterizing different mental disorders have different characteristics and therefore display different vulnerabilities remains unknown.

In summary, psychological networks appear to demonstrate greater complexity than suggested by previous studies. This study concludes that the hypothesis that central and bridge symptoms are good therapeutic targets based solely on a single time-point identification of these symptoms might not be the most fruitful road for network-guided interventions. Moreover, in order to understand the specific processes behind psychological networks, we must explore the full spectrum of network properties and centrality measures that are already available in network science.

## Author Contributions

DC, FF, and TF conceived the study, wrote the initial draft, and edited and revised the manuscript. TF developed the code, prepared the Supplementary Material, and supervised the project. The remaining authors participated in the initial draft of the manuscript and in data collection.

## Conflict of Interest

The authors declare that the research was conducted in the absence of any commercial or financial relationships that could be construed as a potential conflict of interest.

## Supplementary Material

DATA SHEET S1 Plots displaying attack results for each individual network, comparison of the number of modules identified through the Clique Percolation and ModuLand algorithms, and description of the original networks and studies.
