# Supplementary material for: The Differential Role of Central and Bridge Symptoms in Deactivating Psychopathological Networks
Source: Front Psychol. 2019 Nov 1;10:2448. doi: 10.3389/fpsyg.2019.02448 (PMC6849493; doi:10.3389/fpsyg.2019.02448)
Supplement: DATA SHEET S1 — Plots displaying attack results for each individual network, comparison of the number of modules identified through the Clique Percolation and ModuLand algorithms, and description of the original networks and studies. [file Data_Sheet_1.zip › Supplementary.Materials.networks.pdf]

The Differential Role of Central and Bridge Symptoms in Deactivating Psychopathological Networks

Daniel Castro[1,2], Filipa Ferreira[1,2], Inês de Castro[1], Ana Rita Rodrigues[1,2], Marta Correia[1], Josefina Ribeiro[1], Tiago Bento Ferreira[1,2]

[1]University Institute of Maia (Portugal)

[2]Center for Psychology at University of Porto

Supplementary Material

Description of the original networks and studies

Table S1

Description of the original networks and studies

Network Number	Study	Psychopathology	Different Disorders or Problems	Population	Sample	N	Datatype	Supplementary Material	Original Analysis	Analysis	Haslbeck Analysis	Haslbeck N
1	Boschloo et al. (2015)	Mood, Anxiety, and Substance Abuse or Dependence Disorders	Yes	Adults	Community	34653	Binary	Adjacency Matrix	Ising	--	--	--
2	Boschloo et al. (2016)	Internalizing and Externalizing Problems	Yes	Preadolescents	Community	2175	Binary	Adjacency Matrix	Ising	--	--	--
3	Goekoop et al. (2014)	Mood, Anxiety, Schizophrenia, Adjustment, and Personality Disorders	Yes	Adults	Clinical	192	Continuous	Raw Data	Correlations	--	GGM	--
4	Fried et al. (2016)	Depression	No	Adults	Clinical	3463	Continuous	--	GGM		GGM	3463
5	Kendler et al. (2017)	Depression	No	Adults	Clinical	5952	Binary	Adjacency Matrix	Ising	--	--	--
6	Santos et al. (2017).a	Depression	No	Adults	Clinical	264	Continuous	Adjacency Matrix	GGM	--	--	--
7	Santos et al. (2017).b	Depression	No	Adults	Community	240	Continuous	Adjacency Matrix	Ising	--	--	--
8	Armour et al. (2017)	Posttraumatic Stress Disorder	No	Adults	Clinical	221	Continuous	Raw Data	GGM	GGM	--	--
9	Birkeland et al. (2017).a	Posttraumatic Stress Disorder	No	Adults	Community	117	Continuous	Network Data	GGM	--	--	--
10	Birkeland et al. (2017).b	Posttraumatic Stress Disorder	No	Adults	Community	73	Continuous	Network Data	GGM	--	--	--
11	Fried et al. (2018).a	Posttraumatic Stress Disorder	No	Adults	Clinical	526	Continuous	Network Data	GGM	--	--	--

(continue)

Table S1

Description of the original networks and studies (continued)

Network Number	Study	Psychopathology	Different Disorders or Problems	Population	Sample	N	Datatype	Supplementary Material	Original Analysis	Analysis	Haslbeck Analysis	Haslbeck N
12	Fried et al. (2018).b	Posttraumatic Stress Disorder	No	Adults	Clinical	365	Continuous	Network Data	GGM	--	--	--
13	Fried et al. (2018).c	Posttraumatic Stress Disorder	No	Adults	Clinical	926	Continuous	Network Data	GGM	--	--	--
14	Fried et al. (2018).d	Posttraumatic Stress Disorder	No	Adults	Clinical	965	Continuous	Network Data	GGM	--	--	--
15	McNally et al. (2015)	Posttraumatic Stress Disorder	No	Adults	Clinical	362	Continuous	--	Partial Correlations	--	GGM	362
16	McNally et al. (2017)	Posttraumatic Stress Disorder	No	Adults	Clinical	179	Continuous	Raw Data	GGM	GGM	--	--
17	Sullivan et al. (2018)	Posttraumatic Stress Disorder	No	Adults	Community	4639	Continuous	Adjacency Matrix	GGM	--	--	--
18	Anderson et al. (2015)	Autism	No	Children	Clinical	477	Continuous	--	Partial Correlations	--	GGM	477
19	Rhemtulla et al. (2016).a	Substance Abuse	No	Adults	Clinical	2230	Binary	--	Ising	--	Ising	2405
20	Rhemtulla et al. (2016).b	Substance Abuse	No	Adults	Clinical	358	Binary	--	Ising	--	Ising	
21	Rhemtulla et al. (2016).c	Substance Abuse	No	Adults	Clinical	677	Binary	--	Ising	--	Ising	
22	Rhemtulla et al. (2016).d	Substance Abuse	No	Adults	Clinical	639	Binary	--	Ising	--	Ising	

(continue)

Table S1

Description of the original networks and studies (continued)

Network Number	Study	Psychopathology	Different Disorders or Problems	Population	Sample	N	Datatype	Supplementary Material	Original Analysis	Analysis	Haslbeck Analysis	Haslbeck N
23	Rhemtulla et al. (2016).e	Substance Abuse	No	Adults	Clinical	199	Binary	--	Ising	--	Ising	
24	Rhemtulla et al. (2016).f	Substance Abuse	No	Adults	Clinical	350	Binary	--	Ising	--	Ising	
25	Richetin et al. (2017).a	Borderline Personality Disorder	No	Adults	Clinical	96	Continuous	Raw Data	FGM	GGM	--	--
26	Richetin et al. (2017).b	Borderline Personality Disorder	No	Adults	Community	1317	Continuous	Raw Data	FGM	GGM	--	--
27	Koenders et al. (2015).a	Bipolar Disorder	No	Adults	Clinical	47	Continuous	Raw Data	Correlations	--	GGM	126
28	Koenders et al. (2015).b	Bipolar Disorder	No	Adults	Clinical	42	Continuous	Raw Data	Correlations	--	GGM	
29	Koenders et al. (2015).c	Bipolar Disorder	No	Adults	Clinical	36	Continuous	Raw Data	Correlations	--	GGM	
30	Wigman at al. (2016)	Psychosis	Yes	Children	Clinical	283	Binary	--	Ising	--	Ising	283
31	Marcus et al. (2018).a	Dark Personality Traits	Yes	Adults	Community	2831	Continuous	Correlation Matrix	Adaptive Lasso	GGM	--	--
32	Marcus et al. (2018).b	Dark Personality Traits	Yes	Adults	Community	844	Continuous	Correlation Matrix	Adaptive Lasso	GGM	--	--
33	Watters et al. (2016)	Alexithymia	No	Adults	Community	839	Continuous	Adjacency Matrix	Adaptive Lasso	--	--	--

(continue)

Table S1

Description of the original networks and studies (continued)

Network Number	Study	Psychopathology	Different Disorders or Problems	Population	Sample	N	Datatype	Supplementary Material	Original Analysis	Analysis	Haslbeck Analysis	Haslbeck N
34	DuBois et al. (2017)	Eating Disorders	Yes	Adolescents, Adults	Clinical	194	Continuous	Adjacency Matrix	GGM	--	--	--
35	Goldschmidt et al. (2018)	Eating Disorders	Yes	Children, Adolescents	Clinical	636	Continuous	Correlation Matrix	GGM	GGM	--	--
36	Robinaugh et al. (2016)	Grief	No	Adults	Community	195	Continuous	--	GGM	--	GGM	195
37	Robinaugh et al. (2014)	Grief, Depression	Yes	Adults	Community	265	Continuous	--	Correlation	--	GGM	1532
38	Fried et al. (2015)	Grief, Depression	Yes	Adults	Community	515	Binary	--	Ising	--	Ising	515
39	Bellet et al. (2018)	Grief, Posttraumatic Growth	Yes	Adults	Community	485	Continuous	Correlation Matrix	GGM	GGM	--	--
40	Beard et al. (2016)	Depression, Anxiety	Yes	Adults	Clinical	742	Continuous	Covariance Matrix	GGM	GGM	--	--
41	Borsboom et al. (2013).a	Depression, Anxiety	Yes	Adults	Community	9282	Binary	Raw Data	Correlations	Ising	--	--
42	Borsboom et al. (2013).b	Anxiety	No	Adults	Community		Binary	Raw Data	Partial Correlations	Ising	--	--
43	Van Rooijen et al. (2018)	Depression, Psychosis	Yes	Adults	Clinical	470	Continuous	Correlation Matrix	GGM	GGM	--	--
44	Jones et al. (2018)	Depression, Obsessive Compulsive Disorder	Yes	Adolescents	Clinical	87	Continuous	Raw Data	GGM	GGM	--	--

(continue)

Table S1

Description of the original networks and studies (continued)

Network Number	Study	Psychopathology	Different Disorders or Problems	Population	Sample	N	Datatype	Supplementary Material	Original Analysis	Analysis	Haslbeck Analysis	Haslbeck N
45	McNally et al. (2017)	Depression, Obsessive Compulsive Disorder	Yes	Adults	Clinical	408	Continuous	Raw Data	GGM	GGM	--	--
46	Ruzzano et al. (2015)	Autism, Obsessive Compulsive Disorder	Yes	Children	Clinical	213	Binary	--	PC	--	Ising	213
47	Afzali et al. (2017)	PTSD, Alcohol Use	Yes	Adults	Community	449	Binary	Adjacency Matrix	Ising	--	--	--
48	Castro et al. (2018)	Bipolar, Borderline Personality Disorder	Yes	Adults	Community	7556	Binary	Raw Data	Ising	GGM	--	--
49	Bekhuis et al. (2016)	Depression, Anxiety, Somatic Symptoms	Yes	Adults	Clinical	2704	Binary	Adjacency Matrix	Ising	--	--	--
50	Smith et al. (2018)	Eating Disorder, Depression, Anxiety	Yes	Adults	Clinical	446	Continuous	Adjacency Matrix	GGM	--	--	--
51	Marchetti (2018)	Hopelessness	No	Adults	Community	1985	Continuous	Correlation Matrix	GGM	GGM	--	--

References

Afzali, M. H., Sunderland, M., Batterham, P. J., Carragher, N., Calear, A., & Slade, T. (2017). Network approach to the symptom-level association between alcohol use disorder and posttraumatic stress disorder. *Social Psychiatry and Psychiatric Epidemiol*ogy, *52*, 329-339. doi: 10.1007/s00127-016-1331-3

Anderson, M., Montazeri, F., & de Bildt, A. (2015). Network approach to autistic traits: group and subgroup analyses of ADOS item scores. *Journal of Autism and Developmental Disorders*, *45*, 3115-3132. doi: 10.1007/s10803-015-2537-z

Armour, C., Fried, E., Deserno, M. K., Tsai, J., & Pietrzak, H. (2017). A network analysis of DSM-5 posttraumatic stress disorder symptoms and correlates in US military veterans. *Journal of Anxiety Disorders*, *45*, 49-59. doi: 10.1016/j.janxdis.2016.11.008

Beard, C., Millner, A. J., Forgeard, M. J., Fried, E., Hsu, K. J., Treadway, M. T., Leonard, C. V., Kertz, S. J., & Björgvinsson, T. (2016). Network analysis of depression and anxiety symptom relationships in a psychiatric sample. *Psychological Medicine, 46*(16), 3359-3369. doi: 10.1017/S0033291716002300

Bekhuis, E., Schoevers, R., Van Borkulo, D., Rosmalen, M., & Boschloo, L. (2016). The network structure of major depressive disorder generalized anxiety disorder and somatic symptomatology. *Psychological Medicine*, *46*(14), 2989–2998. doi: 10.1017/S0033291716001550

Bellet, W., Jones, P., Neimeyer, A., & McNally, R. (2018). Bereavement outcomes as causal systems: A network analysis of the co-occurrence of complicated grief and posttraumatic growth. *Clinical Psychological Science, 6*(6), 797-809. doi: 10.1177/2167702618777454

Birkeland, S., Blix, I., Solberg, O., & Heir, T. (2017). Gender differences in posttraumatic stress symptoms after a terrorist attack: a network approach. *Frontiers in Psychology, 8*(2091), 1-12. doi: 10.3389/fpsyg.2017.02091

Boschloo, L., Schoevers, R. A., van Borkulo, C. D., Borsboom, D., & Oldehinkel, A. J. (2016). The network structure of psychopathology in a community sample of preadolescents. *Journal of Abnormal Psychology, 125*(4), 599. doi: 10.1037/abn0000150

Boschloo, L., van Borkulo, C. D., Rhemtulla, M., Keyes, K. M., Borsboom, D., & Schoevers, R. A. (2015). The network structure of symptoms of the diagnostic and statistical manual of mental disorders. *PLoS ONE, 10*(9), 1-12. doi: 10.1371/journal.pone.0137621

Castro, D., Ferreira, F., Mendes, A. S., & Ferreira, T. B. (2018). Bridges between bipolar and borderline personality disorders: Clarifying comorbidity through the analysis of the complex network of connections between symptoms. *The Psychologist: Practice & Research Journal, 1*(2).

DuBois, H., Rodgers, F., Franko, L., Eddy, T., & Thomas, J. (2017). A network analysis investigation of the cognitive-behavioral theory of eating disorders. *Behaviour Research and Therapy, 97*, 213-221. doi: 10.1016/j.brat.2017.08.004

Fried, E., Bockting, C., Arjadi, R., Borsboom, D., Amshoff, M., Cramer, A. O., ... & Stroebe, M. (2015). From loss to loneliness: the relationship between bereavement and depressive symptoms. *Journal of Abnormal Psychology, 124*(2), 256-265. doi: 10.1037/abn0000028

Fried, E., Eidhof, B., Palic, S., Costantini, G., Huisman-van Dijk, H. M., Bockting, C. L., ... & Karstoft, K. I. (2018). Replicability and generalizability of posttraumatic stress disorder (PTSD) networks in a cross-cultural multisite study of PTSD symptoms in four trauma patient samples. *Clinical Psychological Science, 6*(3), 335-351. doi: 10.1177/2167702617745092

Fried, E., Epskamp, S., Nesse, R. M. Tuerlinckx, F. & Borsboom, D. (2016). What are good depression symptoms? Comparing the centrality of DSM and non-DSM symptoms of depression in a network analysis. *Journal of Affective Disorders, 189*, 314-320. doi: 10_1016/j_jad_2016_Og_006

Goekoop, R., & Goekoop, J. G. (2014). A network view on psychiatric disorders: Network clusters of symptoms as elementary syndromes of psychopathology. *PLoS ONE, 9*(11). doi: 10.1371/journal.pone.0112734

Goldschmidt, B., Crosby, R. D., Cao, L., Moessner, M., Forbush, K. T., Accurso, E. C., & Le Grange, D. (2018). Network analysis of pediatric eating disorder symptoms in a treatment-seeking, transdiagnostic sample. *Journal of Abnormal Psychology, 127*(2), 251-264. doi: 10.1037/abn0000327

Jones, P., Mair, P., Riemann, B. C., Mugno, B. L., & McNally, R. J. (2018). A network perspective on comorbid depression in adolescents with obsessive-compulsive disorder. *Journal of Anxiety Disorders, 53*, 1-8. doi: 10.1016/j.janxdis.2017.09.008

Kendler, S., Aggen, H., Flint, J., Borsboom, D., & Fried, E. I. (2017). The centrality of DSM and non-DSM depressive symptoms in Han Chinese women with major depression. *Journal of Affective Disorders, 227*, 739-744. doi: 10.1016/j.jad.2017.11.032

Koenders, A., De Kleijn, R., Giltay, E. J., Elzinga, B. M., Spinhoven, P., & Spijker, A. T. (2015). A network approach to bipolar symptomatology in patients with different course types. *PLoS ONE, 10*(10), 0141420. doi: 10.1371/journal.pone.01414201

Marchetti, I. (2018). Hopelessness: A network analysis. *Cognitive Therapy and Research.* Advance online publication. doi: 10.1007/s10608-018-9981-y

Marcus, D., Preszler, J., & Zeigler-Hill, V. (2018). A network of dark personality traits: what lies at the heart of darkness? *Journal of Research in Personality*, *73*, 56–62. doi: 10.1016/j.jrp.2017.11.003

McNally, R. J., Mair, P., Mugno, B. L., & Riemann, B. C. (2017). Co-morbid obsessive-compulsive disorder and depression: A Bayesian network approach. *Psychological Medicine*, *47*(7), 1204–1214. doi: 10.1017/S0033291716003287

McNally, R., Heeren, A., & Robinaugh, D. J. (2017). A Bayesian network analysis of posttraumatic stress disorder symptoms in adults reporting childhood sexual abuse. *European Journal of Psychotraumatology, 8*(3), 1341276. doi: 10.1080/20008198.2017.1341276

McNally, R., Robinaugh, D. J., Wu, G. W. Y., Wang, L., Deserno, M. K., & Borsboom, D. (2015). Mental disorders as causal systems: a network approach to posttraumatic stress disorder. *Clinical Psychological Science*, *3*(6), 836–849. doi: 10.1177/2167702614553230

Rhemtulla, M., Fried, E. I., Aggen, S. H., Tuerlinckx, F., Kendler, K. S., & Borsboom, D. (2016). Network analysis of substance abuse and dependence symptoms. *Drug and Alcohol Dependence, 161*, 230-237. doi:10.1016/j.drugalcdep.2016.02.005

Richetin J, Preti, E., Costantini, G., & De Panfilis C. (2017) The centrality of affective instability and identity in borderline personality disorder: evidence from network analysis. *PLoS ONE 12*(10), 0186695. doi: 10.1371/ journal.pone.0186695

Robinaugh, D., LeBlanc, N., Vuletich, H., & McNally, R. (2014). Network analysis of persistent complex bereavement disorder in conjugally bereaved adults. *Journal of Abnormal Psychology, 123*, 3, 510-522. doi: 10.1037/abn0000002

Robinaugh, J., Millner, J., & McNally, R. (2016). Identifying highly influential nodes in the complicated grief network. *Journal of Abnormal Psychology, 125*(6), 747-757. doi: 10.1037/abn0000181

Ruzzano, L., Borsboom, D., & Geurts, H. M. (2015). Repetitive behaviors in autism and obsessive–compulsive disorder: New perspectives from a network analysis. *Journal of Autism and Developmental Disorders, 45*(1), 192-202. doi: 10.1007/s10803-014-2204-9

Santos, H., Fried, E., Asafu-Adjei, J., & Ruiz, R. J. (2017). Network structure of perinatal depressive symptoms in Latinas: Relationship to stress and reproductive biomarkers. *Research in Nursing & Health, 40*(3), 218-228. doi: 10.1002/nur.21784

Smith, K. E., Crosby, R. D., Wonderlich, S. A., Forbush, K. T., Mason, T. B., & Moessner, M. (2018). Network analysis: An innovative framework for understanding eating disorder psychopathology. *International Journal of Eating Disorders*, *51*(3), 214–222. doi: 10.1002/eat.22836

Sullivan, C. P., Smith, A. J., Lewis, M., & Jones, R. T. (2018). Network analysis of PTSD symptoms following mass violence. *Psychological Trauma: Theory, Research, Practice, and Policy, 10*(1), 58-66. doi: 10.1037/tra0000237

Van Rooijen, G., Isvoranu, A. M., Kruijt, O. H., van Borkulo, C. D., Meijer, C. J., Wigman, J. T., ... & Kahn, R. S. (2018). A state-independent network of depressive, negative and positive symptoms in male patients with schizophrenia spectrum disorders. *Schizophrenia Research, 193*, 232-239. doi: 10.1016/j.schres.2017.07.035

Watters, A., Taylor, J., & Bagby, M. (2016). Illuminating the theoretical components of alexithymia using bifactor modeling and network analysis. *Psychological Assessment, 28*(6), 627-638. doi: 10.1037/pas0000169

Wigman, T., de Vos, S., Wichers, M., van Os, J., & Bartels-Velthuis, A. A. (2016). A transdiagnostic network approach to psychosis. *Schizophrenia Bulletin, 43*(1), 122-132. doi: 10.1093/schbul/sbw095
