# Supplementary material for: The Differential Role of Central and Bridge Symptoms in Deactivating Psychopathological Networks
Source: Front Psychol. 2019 Nov 1;10:2448. doi: 10.3389/fpsyg.2019.02448 (PMC6849493; doi:10.3389/fpsyg.2019.02448)
Supplement: DATA SHEET S1 — Plots displaying attack results for each individual network, comparison of the number of modules identified through the Clique Percolation and ModuLand algorithms, and description of the original networks and studies. [file Data_Sheet_1.zip › Supplementary.Materials.cpmxmod.pdf]

The Differential Role of Central and Bridge Symptoms in Deactivating Psychopathological Networks

Daniel Castro[1,2], Filipa Ferreira[1,2], Inês de Castro[1], Ana Rita Rodrigues[1,2], Marta Correia[1], Josefina Ribeiro[1], Tiago Bento Ferreira[1,2]

[1]University Institute of Maia (Portugal)

[2]Center for Psychology at University of Porto

Supplementary Material

Comparison of the Number of Modules Identified Through the Clique Percolation and Moduland Algorithms

Network Number	Study	Number of Modules				
		ModuLand	Clique Percolation			
			K = 3	K = 4	K = 5	K = 6
1	Boschloo et al. (2015)	29	14	14	15	12
2	Boschloo et al. (2016)	24	16	19	16	2
3	Goekoop et al. (2014)	13	1	-	-	-
4	Fried et al. (2016)	8	5	6	2	3
5	Kendler et al. (2017)	5	3	2	2	-
6	Santos et al. (2017).a	7	6	6	1	-
7	Santos et al. (2017).b	5	6	5	2	1
8	Armour et al. (2017)	8	6	3	3	1
9	Birkeland et al. (2017).a	7	7	5	1	-
10	Birkeland et al. (2017).b	6	4	3	-	-
11	Fried et al. (2018).a	6	4	6	6	2
12	Fried et al. (2018).b	4	3	5	3	1
13	Fried et al. (2018).c	7	3	6	4	3
14	Fried et al. (2018).d	6	5	6	4	4
15	McNally et al. (2015)	6	8	2	-	-

16	McNally et al. (2017)	7	7	5	5	-
17	Sullivan et al. (2018)	4	3	2	-	-
18	Anderson et al. (2015)	3	2	2	-	-
19	Rhemtulla et al. (2016).a	3	3	2	2	2
20	Rhemtulla et al. (2016).b	4	3	2	-	-
21	Rhemtulla et al. (2016).c	3	3	3	3	-
22	Rhemtulla et al. (2016).d	3	2	1	2	-
23	Rhemtulla et al. (2016).e	2	3	1	-	-
24	Rhemtulla et al. (2016).f	1	5	4	-	-
25	Richetin et al. (2017).a	4	1	2	1	-
26	Richetin et al. (2017).b	3	1	2	1	-
27	Koenders et al. (2015).a	2	2	-	-	-
28	Koenders et al. (2015).b	3	1	-	-	-
29	Koenders et al. (2015).c	3	2	-	-	-
30	Wigman at al. (2016)	15	18	2	-	-
31	Marcus et al. (2018).a	3	3	2	2	1
32	Marcus et al. (2018).b	3	3	2	1	2
33	Watters et al. (2016)	5	6	4	3	-

34	DuBois et al. (2017)	3	2	2	1	-
35	Goldschmidt et al. (2018)	7	7	4	3	4
36	Robinaugh et al. (2016)	4	5	-	-	-
37	Robinaugh et al. (2014)	4	5	2	-	-
38	Fried et al. (2015)	5	4	2	2	1
39	Bellet et al. (2018)	9	7	4	3	3
40	Beard et al. (2016)	4	3	3	3	2
41	Borsboom et al. (2013).a	2	3	3	3	2
42	Borsboom et al. (2013).b	2	2	1	-	-
43	Van Rooijen et al. (2018)	8	6	7	4	-
44	Jones et al. (2018)	6	8	1	2	-
45	McNally et al. (2017)	7	8	7	2	1
46	Ruzzano et al. (2015)	4	6	2	-	-
47	Afzali et al. (2017)	10	9	5	-	-
48	Castro et al. (2018)	9	7	6	5	6
49	Bekhuis et al. (2016)	8	7	6	5	5
50	Smith et al. (2018)	12	10	10	14	4
51	Marchetti (2018)	5	4	3	3	3
