# Supplementary material for: The Differential Role of Central and Bridge Symptoms in Deactivating Psychopathological Networks
Source: Front Psychol. 2019 Nov 1;10:2448. doi: 10.3389/fpsyg.2019.02448 (PMC6849493; doi:10.3389/fpsyg.2019.02448)

The Differential Role of Central and Bridge Symptoms in the Disaggregation of Psychopathological Networks

Daniel Castro[1,2], Filipa Ferreira[1,2], Inês de Castro[1], Ana Rita Rodrigues[1,2], Marta Correia[1], Josefina Ribeiro[1], Tiago Bento Ferreira[1,2]

[1]University Institute of Maia (Portugal)

[2]Center for Psychology at University of Porto

Supplementary Material

Plots Displaying Attack Results for Each Individual Network

Boschloo, L., van Borkulo, C. D., Rhemtulla, M., Keyes, K. M., Borsboom, D., & Schoevers, R. A. (2015).
The network structure of symptoms of the diagnostic and statistical manual of mental disorders.
PLoS One, 10(9), e0137621. https://doi.org/10.1371/journal.pone.0137621

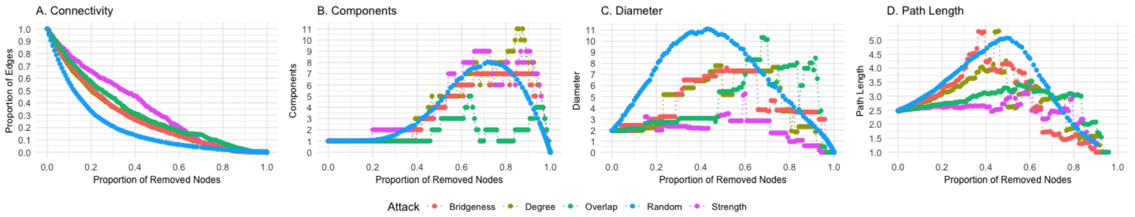

Boschloo, L., Schoevers, R. A., van Borkulo, C. D., Borsboom, D., & Oldehinkel, A. J. (2016).
The network structure of psychopathology in a community sample of preadolescents.
Journal of Abnormal Psychology, 125(4), 599-606. http://dx.doi.org/10.1037/abn0000150

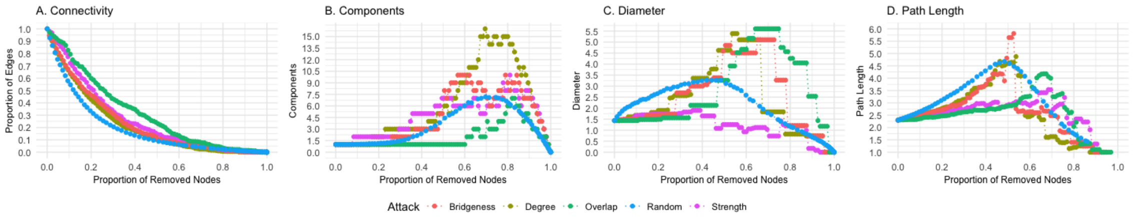

Goekoop, R., & Goekoop, J. G. (2014).
A network view on psychiatric disorders: network clusters of symptoms as elementary syndromes of psychopathology.
PloS one, 9(11), e112734.https://doi.org/10.1371/journal.pone.0112734

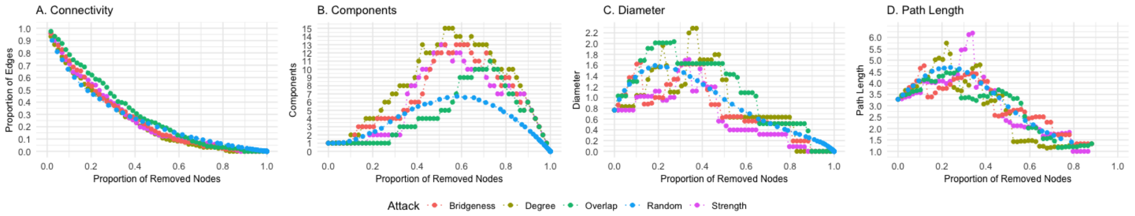

Fried, E. I., Epskamp, S., Nesse, R. M., Tuerlinckx, F., & Borsboom, D. (2016).
What are 'good' depression symptoms? Comparing the centrality of DSM and non-DSM symptoms of depression in a network analysis.
Journal of Affetive Disorders, 189, 314-320. https://doi.org/10.1016/j.jad.2015.09.005

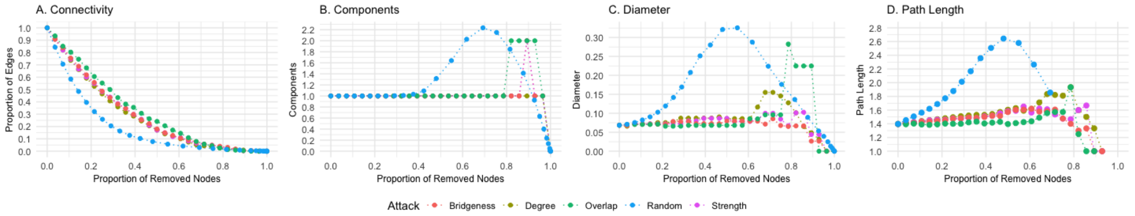

Kendler, K. S., Aggen, S. H., Flint, J., Borsboom, D., & Fried, E. I. (2017).
The centrality of DSM and non-DSM depressive symptoms in Han Chinese women with major depression.
Journal of Affective Disorders, 227, 739-744. https://doi.org/10.1016/j.jad.2017.11.032

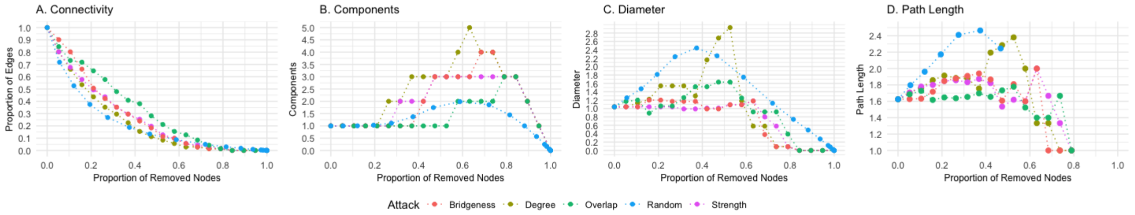

Santos Jr, H., Fried, E. I., Asafu□Adjei, J., & Ruiz, R. J. (2017).
Network structure of perinatal depressive symptoms in Latinas: relationship to stress and reproductive biomarkers.
Research in Nursing & Health, 40(3), 218-228.https://doi.org/10.1002/nur.21784

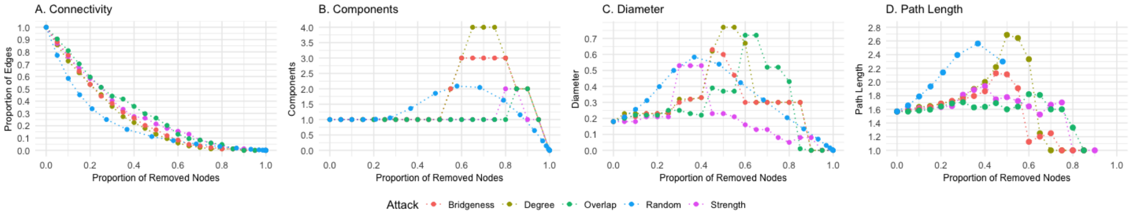

Santos Jr, H., Fried, E. I., Asafu□Adjei, J., & Ruiz, R. J. (2017).
Network structure of perinatal depressive symptoms in Latinas: relationship to stress and reproductive biomarkers.
Research in Nursing & Health, 40(3), 218-228.https://doi.org/10.1002/nur.21784

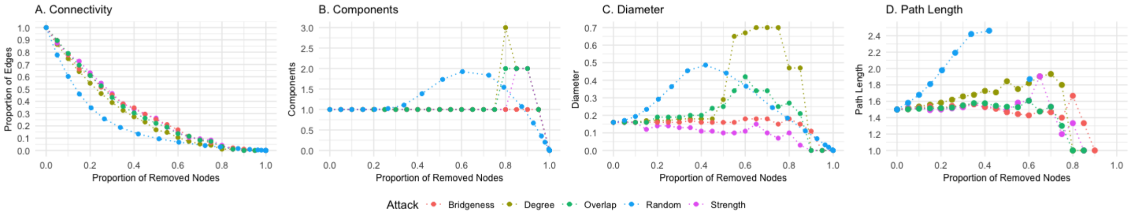

Armour, C., Fried, E. I., Deserno, M. K., Tsai, J., & Pietrzak, R. H. (2017).
A network analysis of DSM-5 posttraumatic stress disorder symptoms and correlates in US military veterans.
Journal of Anxiety Disorders, 45, 49-59. https://doi.org/10.1016/j.janxdis.2016.11.008

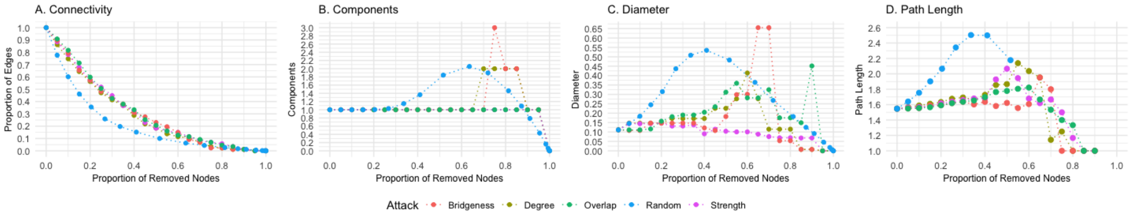

Birkeland, M. S., Blix, I., Solberg, ?., & Heir, T. (2017).
Gender differences in posttraumatic stress symptoms after a terrorist attack: a network approach.
Frontiers in Psychology, 8, 2091. https://doi.org/10.3389/fpsyg.2017.02091

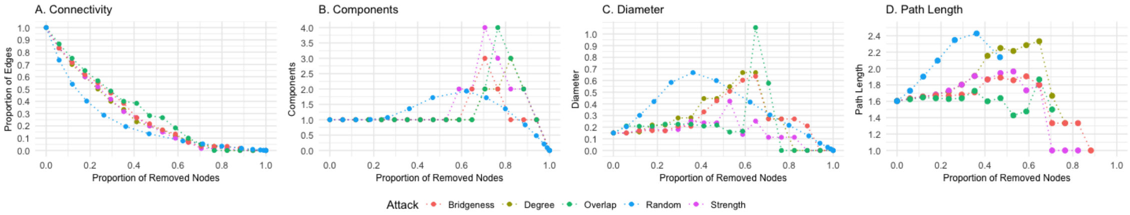

Birkeland, M. S., Blix, I., Solberg, ?., & Heir, T. (2017).
Gender differences in posttraumatic stress symptoms after a terrorist attack: a network approach.
Frontiers in Psychology, 8, 2091. https://doi.org/10.3389/fpsyg.2017.02091

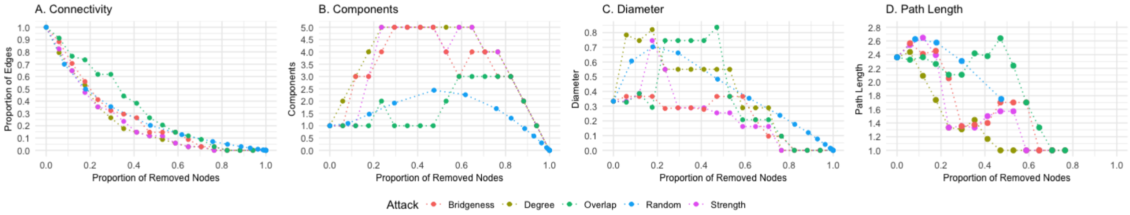

Fried, E. I., Eidhof, M. B., Palic, S., Costantini, G., Huisman-van Dijk, H. M., Bockting, C. L., ... & Karstoft, K. I. (2018).
Replicability and generalizability of posttraumatic stress disorder (PTSD) networks:
a cross-cultural multisite study of PTSD symptoms in four trauma patient samples.
Clinical Psychological Science, 6(3), 335-351. https://doi.org/10.1177/2167702617745092

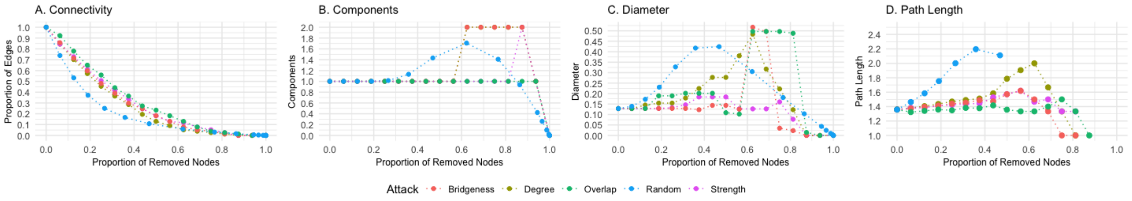

Fried, E. I., Eidhof, M. B., Palic, S., Costantini, G., Huisman-van Dijk, H. M., Bockting, C. L., ... & Karstoft, K. I. (2018).
Replicability and generalizability of posttraumatic stress disorder (PTSD) networks:
a cross-cultural multisite study of PTSD symptoms in four trauma patient samples.
Clinical Psychological Science, 6(3), 335-351. https://doi.org/10.1177/2167702617745092

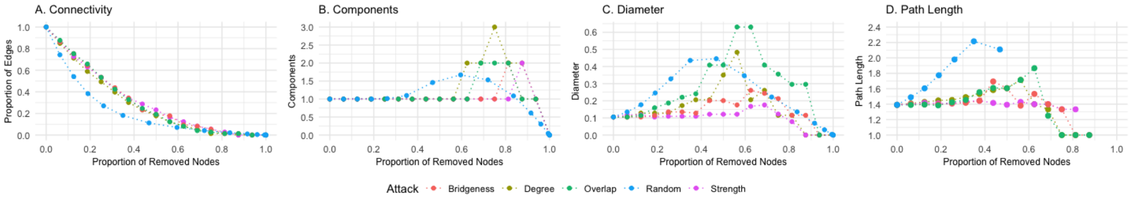

Fried, E. I., Eidhof, M. B., Palic, S., Costantini, G., Huisman-van Dijk, H. M., Bockting, C. L., ... & Karstoft, K. I. (2018).
Replicability and generalizability of posttraumatic stress disorder (PTSD) networks:
a cross-cultural multisite study of PTSD symptoms in four trauma patient samples.
Clinical Psychological Science, 6(3), 335-351. https://doi.org/10.1177/2167702617745092

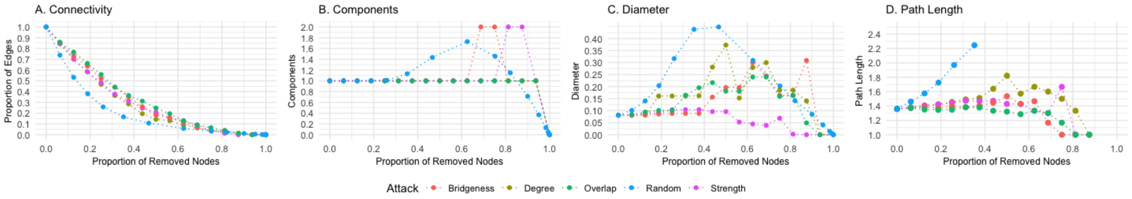

Fried, E. I., Eidhof, M. B., Palic, S., Costantini, G., Huisman-van Dijk, H. M., Bockting, C. L., ... & Karstoft, K. I. (2018).
Replicability and generalizability of posttraumatic stress disorder (PTSD) networks:
a cross-cultural multisite study of PTSD symptoms in four trauma patient samples.
Clinical Psychological Science, 6(3), 335-351. https://doi.org/10.1177/2167702617745092

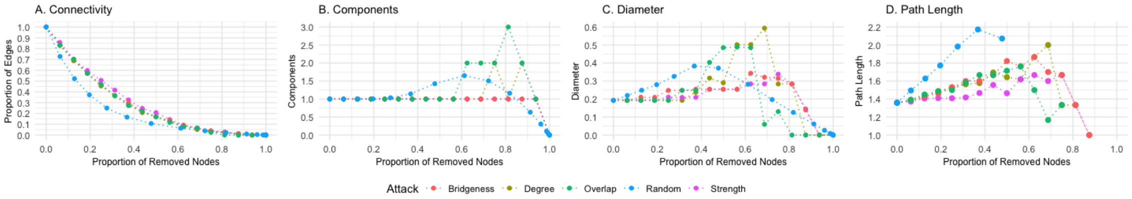

McNally, R. J., Robinaugh, D. J., Wu, G. W., Wang, L., Deserno, M. K., & Borsboom, D. (2015).
Mental disorders as causal systems: A network approach to posttraumatic stress disorder.
Clinical Psychological Science, 3(6), 836-849. https://doi.org/10.1177/2167702614553230

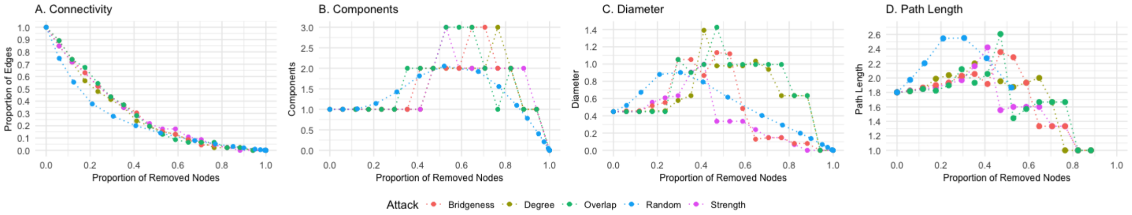

McNally, R. J., Heeren, A., & Robinaugh, D. J. (2017).
A Bayesian network analysis of posttraumatic stress disorder symptoms in adults reporting childhood sexual abuse.
European Journal of Psychotraumatology, 8(sup3), 1341276.https://doi.org/10.1080/20008198.2017.1341276

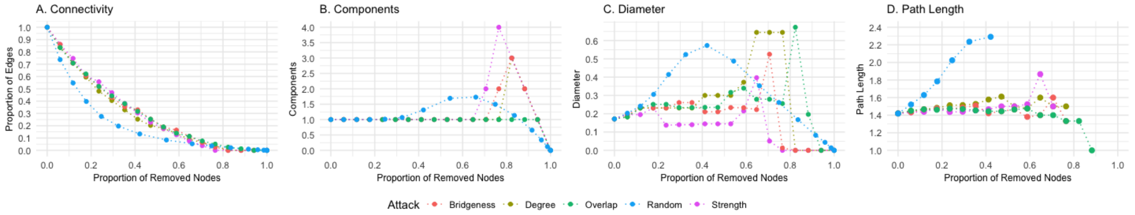

Sullivan, C. P., Smith, A. J., Lewis, M., & Jones, R. T. (2018).
Network analysis of PTSD symptoms following mass violence.
Psychological Trauma: Theory, Research, Practice, and Policy, 10(1), 58-66. http://dx.doi.org/10.1037/tra0000237

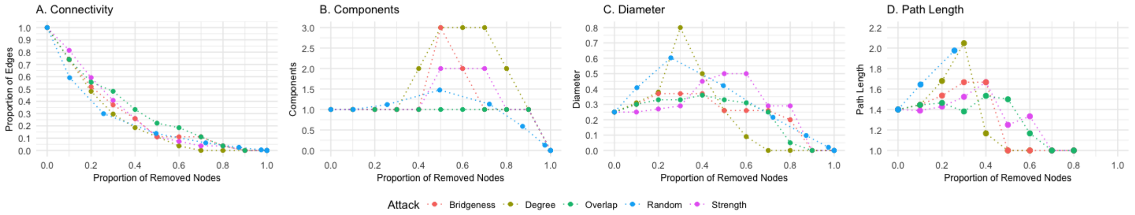

Anderson, G. M., Montazeri, F., & de Bildt, A. (2015).
Network approach to autistic traits: group and subgroup analyses of ADOS item scores.
Journal of Autism and Developmental Disorders, 45(10), 3115-3132. https://doi.org/10.1007/s10803-015-2537-z

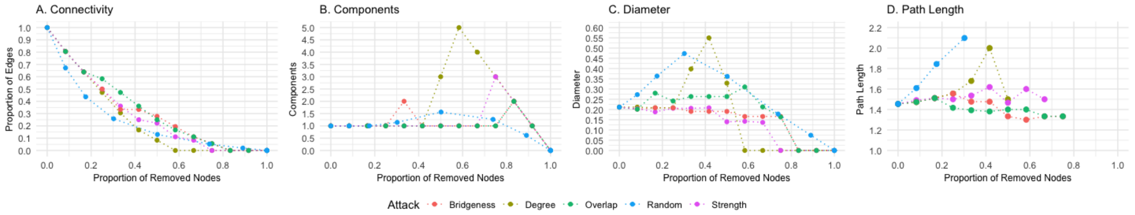

Rhemtulla, M., Fried, E. I., Aggen, S. H., Tuerlinckx, F., Kendler, K. S., & Borsboom, D. (2016).
Network analysis of substance abuse and dependence symptoms.
Drug and Alcohol Dependence, 161, 230-237. https://doi.org/10.1016/j.drugalcdep.2016.02.005

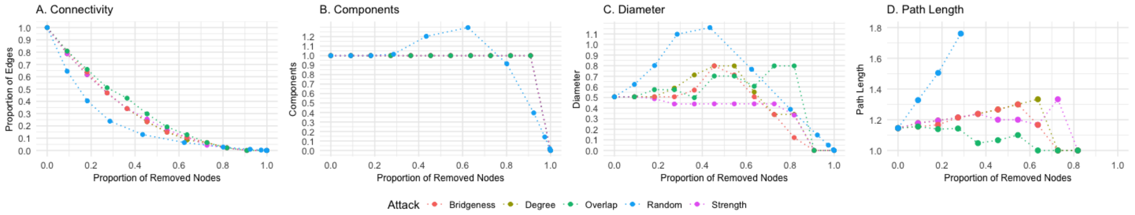

Rhemtulla, M., Fried, E. I., Aggen, S. H., Tuerlinckx, F., Kendler, K. S., & Borsboom, D. (2016).
Network analysis of substance abuse and dependence symptoms.
Drug and Alcohol Dependence, 161, 230-237. https://doi.org/10.1016/j.drugalcdep.2016.02.005

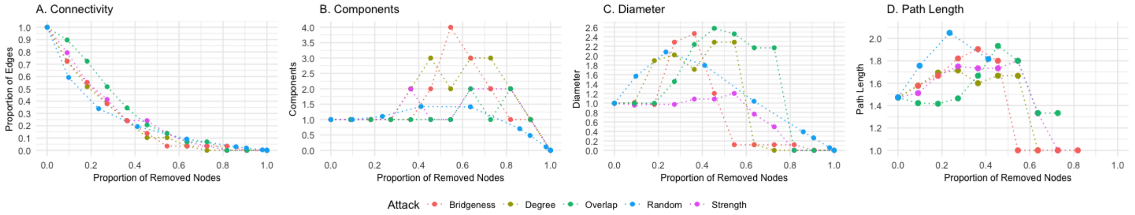

Rhemtulla, M., Fried, E. I., Aggen, S. H., Tuerlinckx, F., Kendler, K. S., & Borsboom, D. (2016).
Network analysis of substance abuse and dependence symptoms.
Drug and Alcohol Dependence, 161, 230-237. https://doi.org/10.1016/j.drugalcdep.2016.02.005

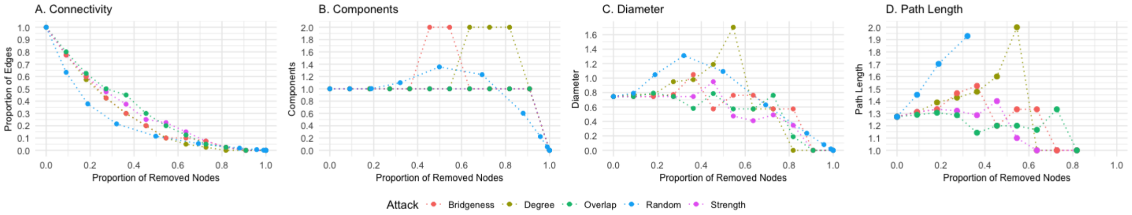

Rhemtulla, M., Fried, E. I., Aggen, S. H., Tuerlinckx, F., Kendler, K. S., & Borsboom, D. (2016).
Network analysis of substance abuse and dependence symptoms.
Drug and Alcohol Dependence, 161, 230-237. https://doi.org/10.1016/j.drugalcdep.2016.02.005

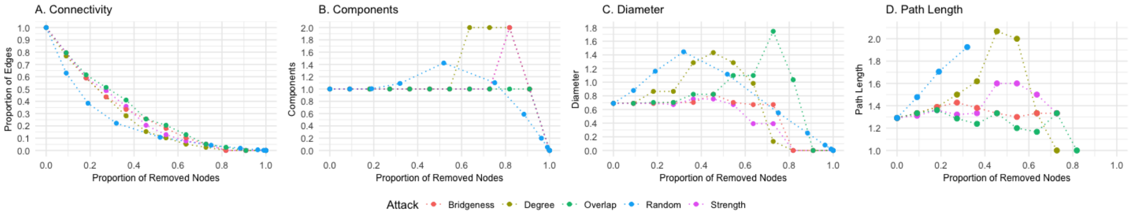

Rhemtulla, M., Fried, E. I., Aggen, S. H., Tuerlinckx, F., Kendler, K. S., & Borsboom, D. (2016).
Network analysis of substance abuse and dependence symptoms.
Drug and Alcohol Dependence, 161, 230-237. https://doi.org/10.1016/j.drugalcdep.2016.02.005

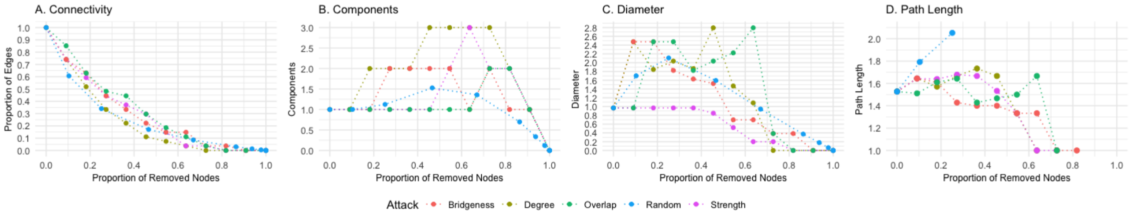

Rhemtulla, M., Fried, E. I., Aggen, S. H., Tuerlinckx, F., Kendler, K. S., & Borsboom, D. (2016).
Network analysis of substance abuse and dependence symptoms.
Drug and Alcohol Dependence, 161, 230-237. https://doi.org/10.1016/j.drugalcdep.2016.02.005

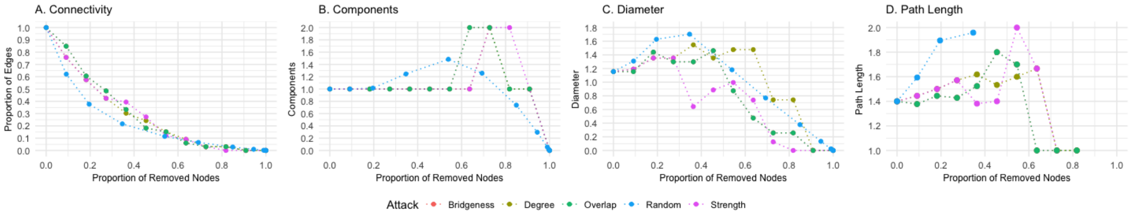

Richetin J, Preti E, Costantini G, De Panfilis C (2017).
The centrality of affective instability and identity in Borderline Personality Disorder: Evidence from network analysis.
PLoS ONE 12(10): e0186695. https://doi.org/10.1371/ journal.pone.0186695

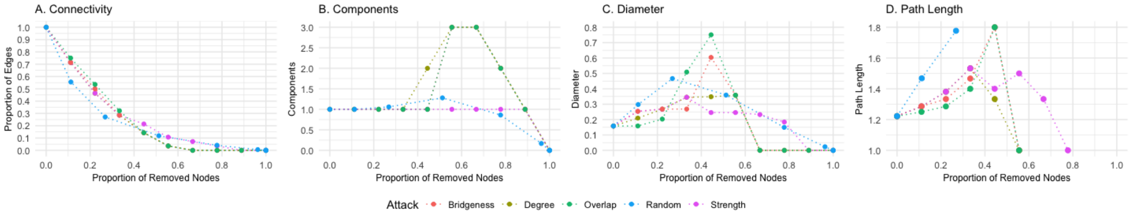

Richetin J, Preti E, Costantini G, De Panfilis C (2017).
The centrality of affective instability and identity in Borderline Personality Disorder: Evidence from network analysis.
PLoS ONE 12(10): e0186695. https://doi.org/10.1371/ journal.pone.0186695

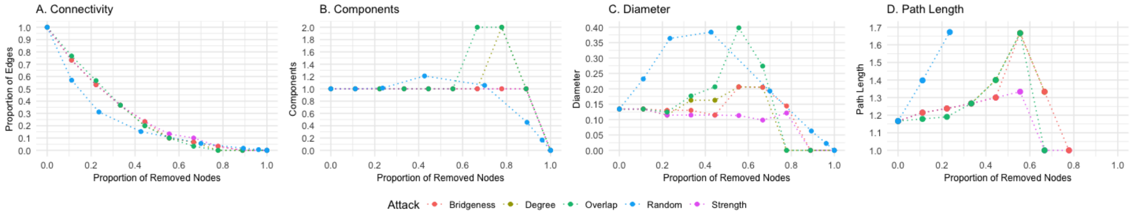

Koenders, M. A., De Kleijn, R., Giltay, E. J., Elzinga, B. M., Spinhoven, P., & Spijker, A. T. (2015).
A network approach to bipolar symptomatology in patients with different course types.
PLoS One, 10(10), e0141420. https://doi.org/10.1371/journal.pone.0141420

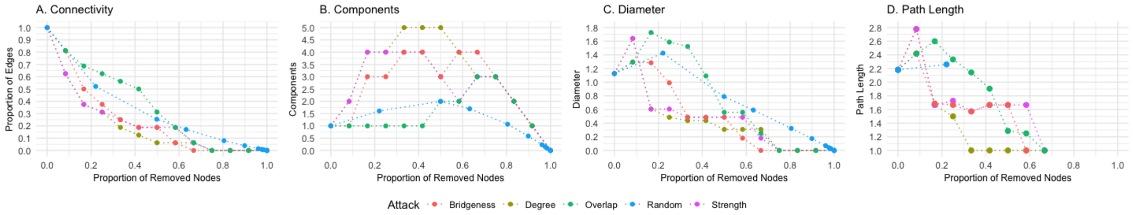

Koenders, M. A., De Kleijn, R., Giltay, E. J., Elzinga, B. M., Spinhoven, P., & Spijker, A. T. (2015).
A network approach to bipolar symptomatology in patients with different course types.
PLoS One, 10(10), e0141420. https://doi.org/10.1371/journal.pone.0141420

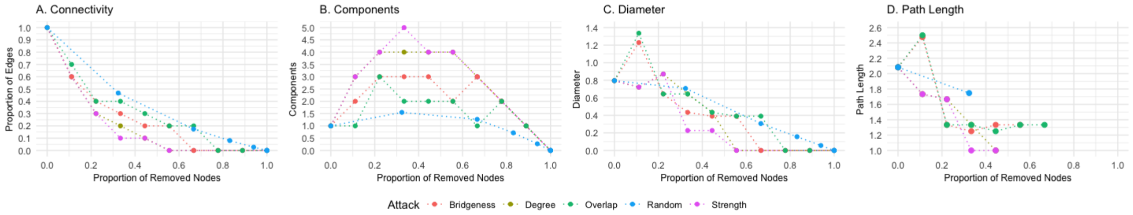

Koenders, M. A., De Kleijn, R., Giltay, E. J., Elzinga, B. M., Spinhoven, P., & Spijker, A. T. (2015).
A network approach to bipolar symptomatology in patients with different course types.
PLoS One, 10(10), e0141420. https://doi.org/10.1371/journal.pone.0141420

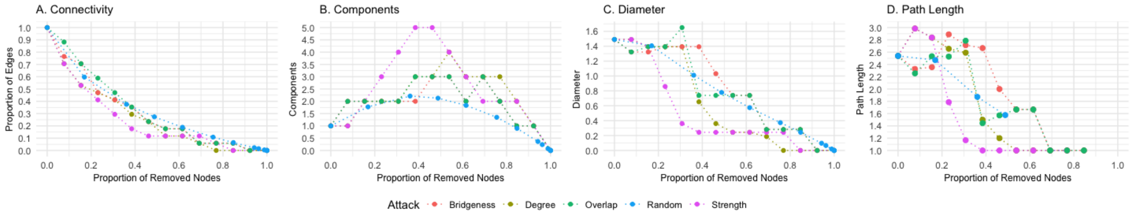

Marchetti, I. (2018).
Hopelessness: A network analysis.
Cognitive Therapy and Research. Advance online publication. https://doi.org/10.1007/s10608-018-9981-y

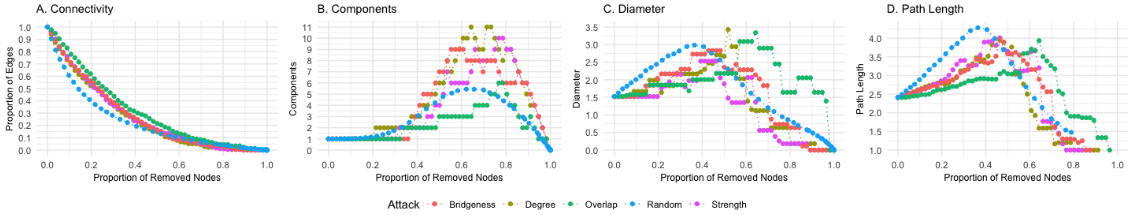

Marcus, D. K., Preszler, J., & Zeigler-Hill, V. (2018).
A network of dark personality traits: What lies at the heart of darkness?.
Journal of Research in Personality, 73, 56-62. https://doi.org/10.1016/j.jrp.2017.11.003

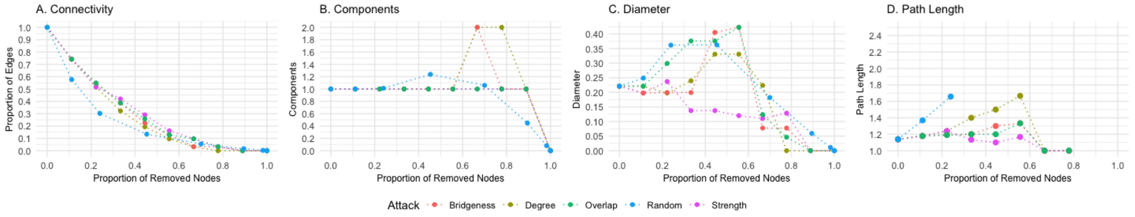

Marcus, D. K., Preszler, J., & Zeigler-Hill, V. (2018).
A network of dark personality traits: What lies at the heart of darkness?.
Journal of Research in Personality, 73, 56-62. https://doi.org/10.1016/j.jrp.2017.11.003

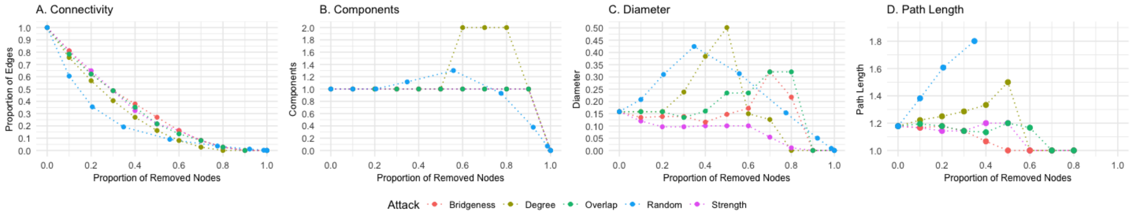

Watters, C. A., Taylor, G. J., & Bagby, R. M. (2016).
Illuminating the theoretical components of alexithymia using bifactor modeling and network analysis.
Psychological Assessment, 28(6), 627-638. http://dx.doi.org/10.1037/pas0000169

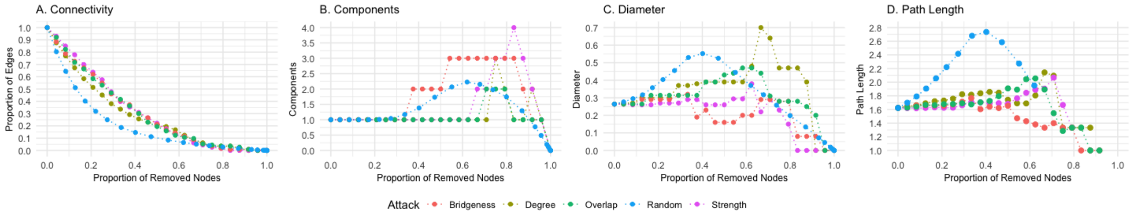

DuBois, R. H., Rodgers, R. F., Franko, D. L., Eddy, K. T., & Thomas, J. J. (2017).
A network analysis investigation of the cognitive-behavioral theory of eating disorders.
Behaviour Research and Therapy, 97, 213-221. http://dx.doi.org/10.1016/j.brat.2017.08.004

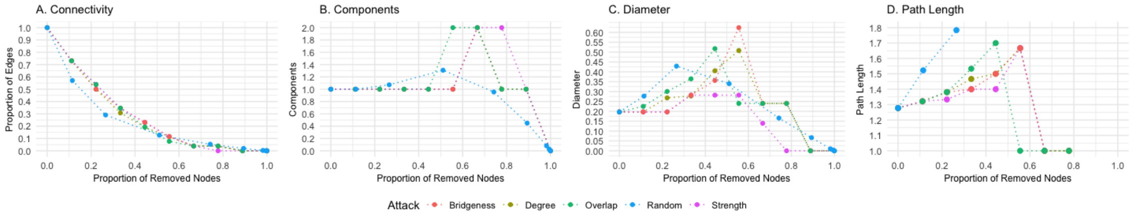

Goldschmidt, A. B., Crosby, R. D., Cao, L., Moessner, M., Forbush, K. T., Accurso, E. C., & Le Grange, D. (2018).
Network analysis of pediatric eating disorder symptoms in a treatment-seeking, transdiagnostic sample.
Journal of Abnormal Psychology, 127(2), 251-264. http://dx.doi.org/10.1037/abn0000327

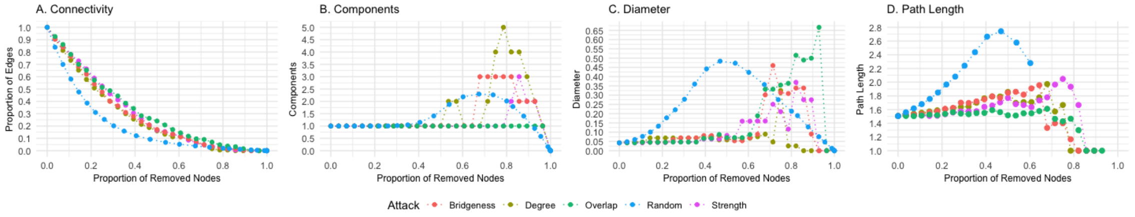

Robinaugh, D. J., Millner, A. J., & McNally, R. J. (2016).
Identifying highly influential nodes in the complicated grief network.
Journal of Abnormal Psychology, 125(6), 747-757. http://dx.doi.org/10.1037/abn0000181

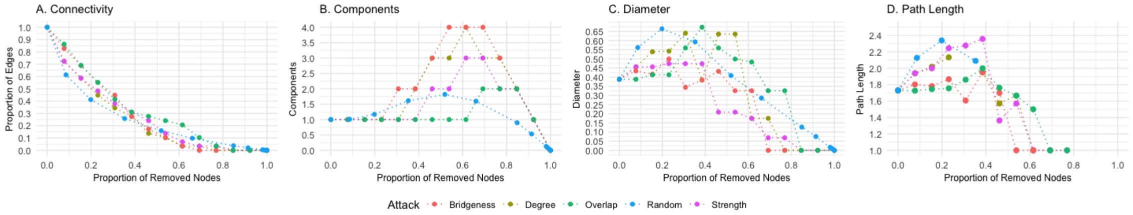

Robinaugh, D. J., LeBlanc, N. J., Vuletich, H. A., & McNally, R. J. (2014).
Network analysis of persistent complex bereavement disorder in conjugally bereaved adults.
Journal of Abnormal Psychology, 123(3), 510-522. http://dx.doi.org/10.1037/abn0000002

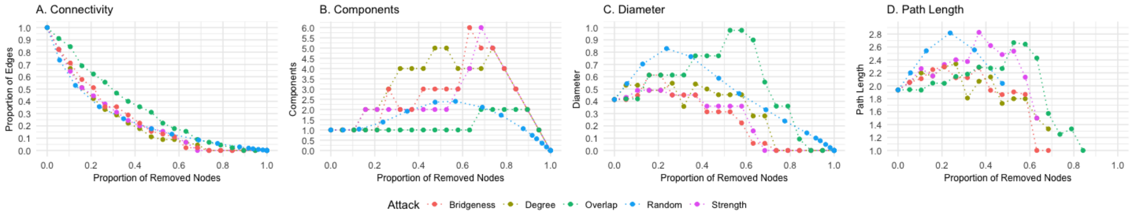

Fried, E. I., Bockting, C., Arjadi, R., Borsboom, D., Amshoff, M., Cramer, A. O., ... & Stroebe, M. (2015).
From loss to loneliness: The relationship between bereavement and depressive symptoms.
Journal of Abnormal Psychology, 124(2), 256-265. http://dx.doi.org/10.1037/abn0000028

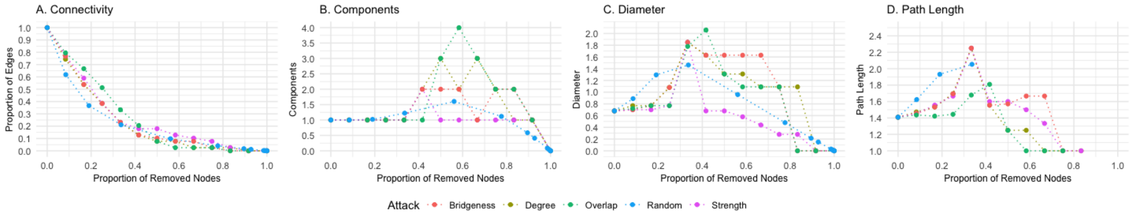

Bellet, B. W., Jones, P. J., Neimeyer, R. A., & McNally, R. J. (2018).
Bereavement Outcomes as Causal Systems: A Network Analysis of the Co-Occurrence of Complicated Grief and Posttraumatic Growth.
Clinical Psychological Science, 6(6), 797-809.https://doi.org/10.1177/2167702618777454

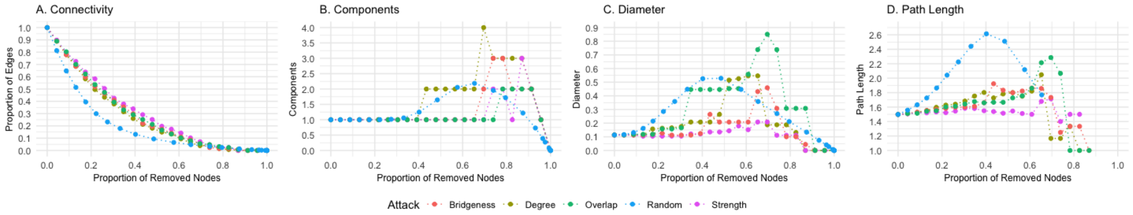

Beard, C., Millner, A. J., Forgeard, M. J., Fried, E. I., Hsu, K. J., Treadway, M. T., ... & Björgvinsson, T. (2016).
Network analysis of depression and anxiety symptom relationships in a psychiatric sample.
Psychological Medicine, 46(16), 3359-3369. https://doi.org/10.1017/S0033291716002300

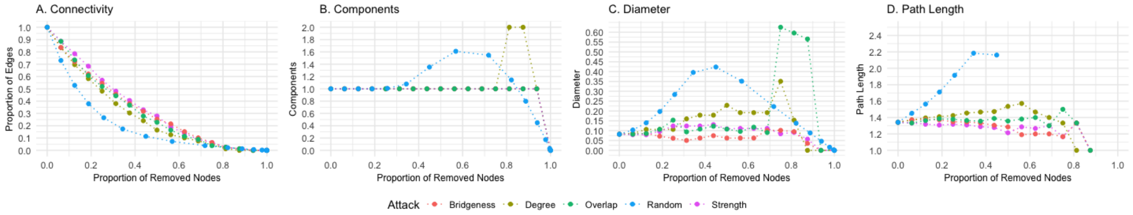

Borsboom, D., & Cramer, A. O. (2013).
Network analysis: an integrative approach to the structure of psychopathology.
Annual Review of Clinical Psychology, 9, 91-121. https://doi.org/10.1146/annurev-clinpsy-050212-185608

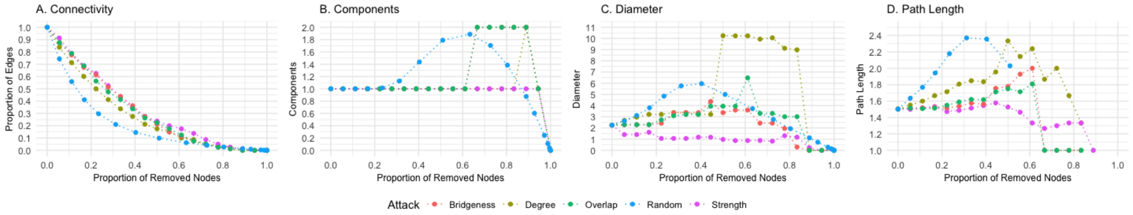

Borsboom, D., & Cramer, A. O. (2013).
Network analysis: an integrative approach to the structure of psychopathology.
Annual Review of Clinical Psychology, 9, 91-121. https://doi.org/10.1146/annurev-clinpsy-050212-185608

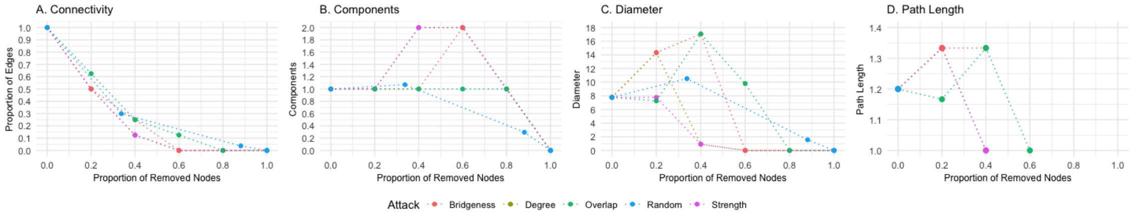

van Rooijen, G., Isvoranu, A. M., Kruijt, O. H., van Borkulo, C. D., Meijer, C. J., Wigman, J. T., ... & Kahn, R. S. (2018).
A state-independent network of depressive, negative and positive symptoms in male patients with schizophrenia spectrum disorders.
Schizophrenia Research, 193, 232-239. http://dx.doi.org/10.1016/j.schres.2017.07.035

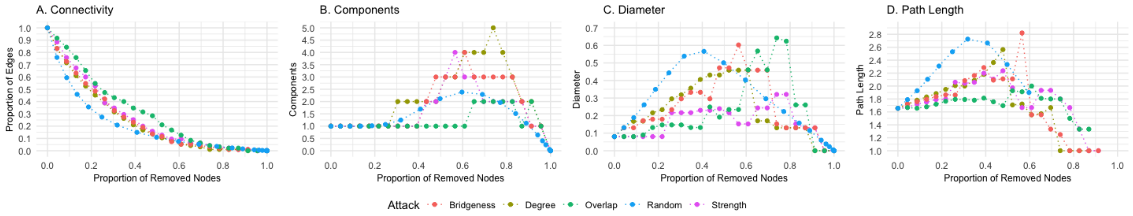

Jones, P. J., Mair, P., Riemann, B. C., Mugno, B. L., & McNally, R. J. (2018).
A network perspective on comorbid depression in adolescents with obsessive-compulsive disorder.
Journal of Anxiety Disorders, 53, 1-8. https://doi.org/10.1016/j.janxdis.2017.09.008

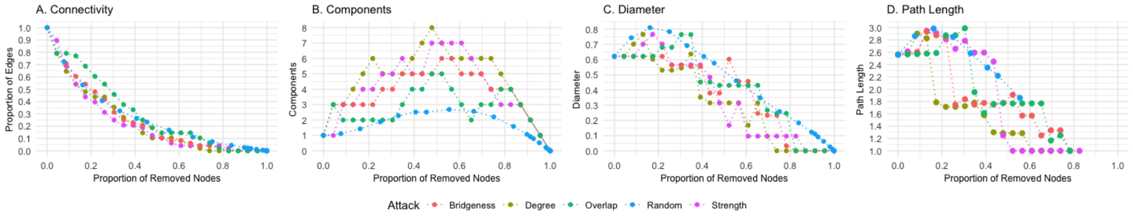

McNally, R. J., Mair, P., Mugno, B. L., & Riemann, C. (2017).
Co-morbid obsessive-compulsive disorder and depression: a bayesian network approach.
Psychological Medicine. 47, 1204-1214. doi:10.1017/S0033291716003287

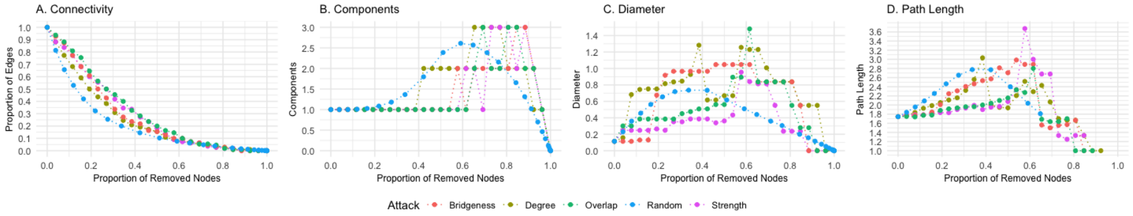

Ruzzano, L., Borsboom, D., & Geurts, H. M. (2015).
Repetitive behaviors in autism and obsessive–compulsive disorder: New perspectives from a network analysis.
Journal of Autism and Developmental Disorders, 45(1), 192-202. https://doi.org/10.1007/s10803-014-2204-9

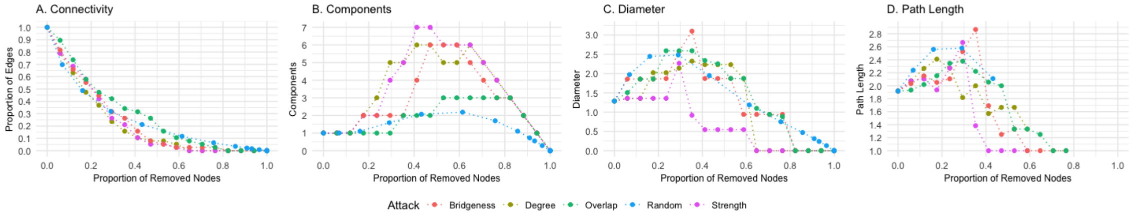

Afzali, M. H., Sunderland, M., Batterham, P. J., Carragher, N., Calear, A., & Slade, T. (2017).
Network approach to the symptom-level association between alcohol use disorder and posttraumatic stress disorder.
Social Psychiatry and Psychiatric Epidemiology, 52(3), 329-339. https://doi.org/10.1007/s00127-016-1331-3

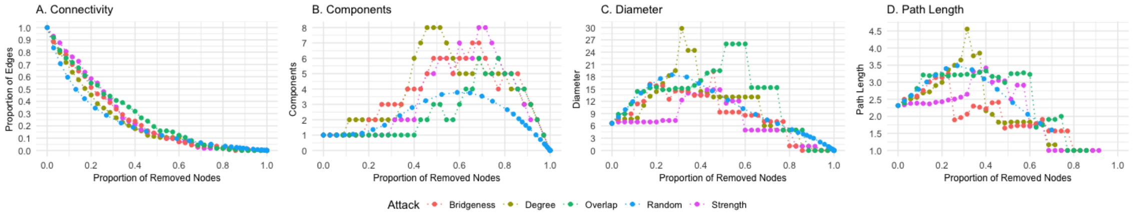

Castro, D., Ferreira, F., Mendes, A. S., & Ferreira, T. B. (2018).
Bridges Between Bipolar and Borderline Personality Disorders: Clarifying Comorbidity Through the Analysis of the Complex Network of Connections Between Symptoms.
The Psychologist: Practice & Research Journal, 1(2).

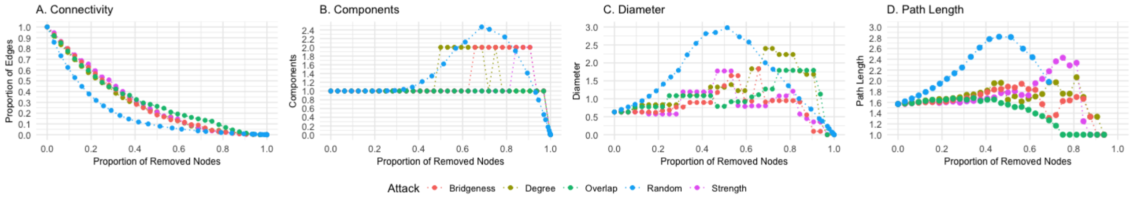

Bekhuis, E., Schoevers, R. A., van Borkulo, C. D., Rosmalen, J. G. M., & Boschloo, L. (2016).
The network structure of major depressive disorder, generalized anxiety disorder and somatic symptomatology.
Psychological Medicine, 46(14), 2989-2998. https://doi.org/10.1017/S0033291716001550

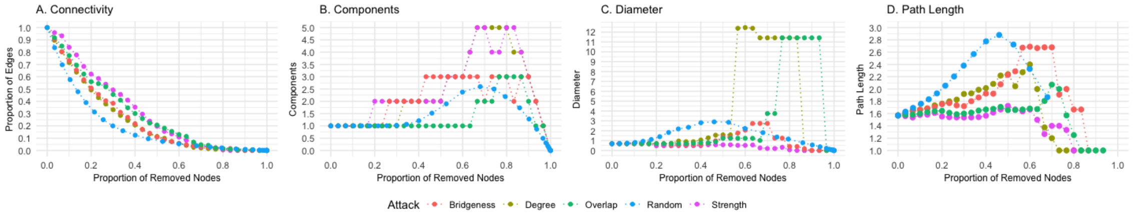

Smith, K. E., Mason, T. B., Crosby, R. D., Cao, L., Leonard, R. C., Wetterneck, C. T., ... & Moessner, M. (2018).
A comparative network analysis of eating disorder psychopathology and co-occurring depression and anxiety symptoms before and after treatment.
Psychological Medicine. Advance online publication. https://doi.org/10.1017/S0033291718000867

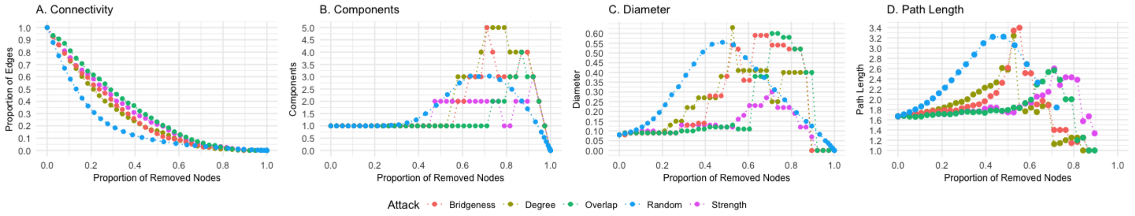

Marchetti, I. (2018).
Hopelessness: A network analysis.
Cognitive Therapy and Research. Advance online publication. https://doi.org/10.1007/s10608-018-9981-y

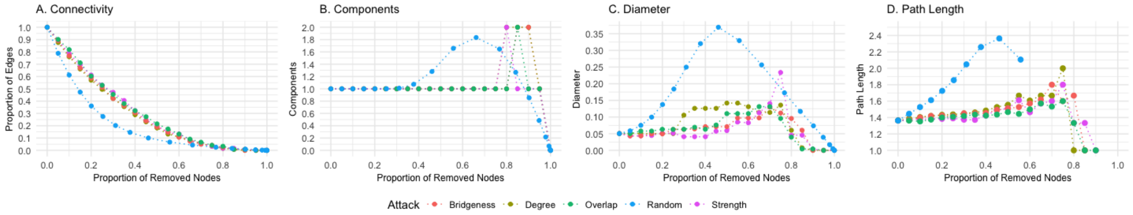

Supplement: DATA SHEET S1 — Plots displaying attack results for each individual network, comparison of the number of modules identified through the Clique Percolation and ModuLand algorithms, and description of the original networks and studies. [file Data_Sheet_1.zip › Supplementary.Materials.attackPlots.pdf]
